# Thiophene-Based Ligands for Specific Assignment of Distinct Aβ Pathologies in Alzheimer's Disease

**DOI:** 10.1021/acschemneuro.4c00021

**Published:** 2024-03-25

**Authors:** Therése Klingstedt, Linda Lantz, Hamid Shirani, Junyue Ge, Jörg Hanrieder, Ruben Vidal, Bernardino Ghetti, K. Peter R. Nilsson

**Affiliations:** † Department of Physics, Chemistry and Biology, Linköping University, Linköping 581 83, Sweden; ‡ Department of Psychiatry and Neurochemistry, Institute of Neuroscience and Physiology, The Sahlgrenska Academy, University of Gothenburg, Mölndal Hospital, Mölndal 431 80, Sweden; § Department of Neurodegenerative Diseases, University College London Institute of Neurology, Queen Square, London WC1N 3BG, United Kingdom; ∥ Department of Pathology and Laboratory Medicine, Indiana University School of Medicine, Indianapolis, Indiana 46202, United States

**Keywords:** Alzheimer’s disease, amyloid-β, protein aggregates, ligands, fluorescence, imaging mass spectrometry

## Abstract

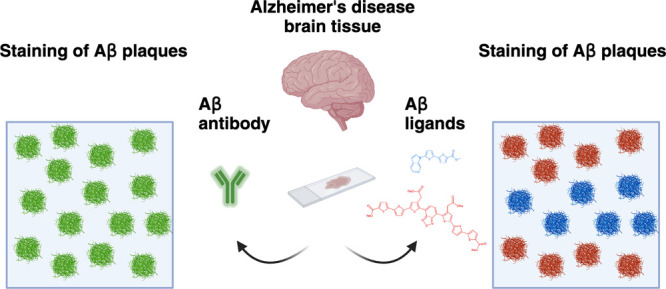

Aggregated species of amyloid-β (Aβ) are one of the pathological hallmarks in Alzheimer’s disease (AD), and ligands that selectively target different Aβ deposits are of great interest. In this study, fluorescent thiophene-based ligands have been used to illustrate the features of different types of Aβ deposits found in AD brain tissue. A dual-staining protocol based on two ligands, HS-276 and LL-1, with different photophysical and binding properties, was developed and applied on brain tissue sections from patients affected by sporadic AD or familial AD associated with the *PSEN1 A431E* mutation. When binding to Aβ deposits, the ligands could easily be distinguished for their different fluorescence, and distinct staining patterns were revealed for these two types of AD. In sporadic AD, HS-276 consistently labeled all immunopositive Aβ plaques, whereas LL-1 mainly stained cored and neuritic Aβ deposits. In the *PSEN1 A431E* cases, each ligand was binding to specific types of Aβ plaques. The ligand-labeled Aβ deposits were localized in distinct cortical layers, and a laminar staining pattern could be seen. Biochemical characterization of the Aβ aggregates in the individual layers also showed that the variation of ligand binding properties was associated with certain Aβ peptide signatures. For the *PSEN1 A431E* cases, it was concluded that LL-1 was binding to cotton wool plaques, whereas HS-276 mainly stained diffuse Aβ deposits. Overall, our findings showed that a combination of ligands was essential to identify distinct aggregated Aβ species associated with different forms of AD.

## Introduction

Extracellular plaques formed by filaments of the amyloid-β (Aβ) peptide and intraneuronal neurofibrillary tangles (NFTs) made of insoluble hyperphosphorylated tau are two pathological hallmarks of Alzheimer’s disease (AD). In the Aβ deposits, different species of Aβ peptides can be found. The 40 and 42 amino acid-long variants Aβ1-40 and Aβ1-42 are the most investigated forms of which the latter is the most abundant peptide in the filaments of parenchymal plaques whereas the former is found in vascular deposits.^1−6^ By studying aggregates prepared from recombinant or synthetic Aβ peptides, evidence that Aβ filaments exhibit a conformational heterogeneity first emerged.^7−12^ To investigate if the results in vitro truly reflected the pathologic properties of Aβ in vivo, experiments on Aβ filaments extracted from human AD brains have been performed. Solid-state nuclear magnetic resonance measurements on Aβ filaments derived by seeded growth from brain tissue of AD patients with different disease phenotypes or from individuals with distinct AD clinical subtypes have shown a structural variability.^13,14^ More recently, by using cryogenic electron microscopy (cryo-EM), high-resolution structures of Aβ1-40 or Aβ1-42 filaments isolated from the vasculature of the meninges or the cortex of the brains of AD patients have been reported.^15,16^ The results showed that Aβ1-40 and Aβ1-42 filaments were structurally different and that filaments formed from each peptide exhibited a conformational heterogeneity. Moreover, the structural properties of brain-derived and in vitro formed Aβ filaments differed.^15,16^

The processes resulting in the formation of Aβ plaques and NFTs occur early in the disease pathogenesis;^17−21^ therefore, the development of imaging ligands that detect these pathological lesions in living subjects would allow for an earlier diagnosis as well as aid in the studies of the pathogenesis of the disease. Pittsburgh compound-B (PiB) was the first ligand to be used clinically as a positron emission tomography (PET) tracer for Aβ deposits^22^ and is still used for this application. However, occasionally, the highest retention of PiB shows a weak correlation with the brain area identified as having the greatest burden of Aβ lesions.^22^ Furthermore, the ligand binds poorly to Aβ deposits in certain brain regions^23,24^ and has been reported to show substantially reduced binding to brain homogenate from AD patient despite evidence of heavy Aβ load.^25^ Recently, it was reported that PiB has a limited ability to bind to filaments in cotton wool plaques (CWPs).^26^ CWPs are made of Aβ; they are round and histologically well-demarcated and lack a dense core. They are found most frequently in cases of genetically determined AD in association with a subset of presenilin-1 (*PSEN1*) mutations.^26−28^ Since CWPs are abundant in association with this subset of genetically determined AD, decreased binding of PiB to CWPs results in an underestimation of the load of Aβ deposits.^26^

The variation in PiB binding displayed by Aβ plaques argues for the need of additional ligands for the detection of a variety of aggregated Aβ. In this regard, fluorescent thiophene-based ligands, especially the family of luminescent conjugated oligothiophenes (LCOs), have been used to detect and study a variety of protein aggregates in vitro, in tissue samples, and in vivo.^29−48^ The binding properties of the LCOs are highly dependent on the chemical structure of the ligand, for example, to achieve detection of early formed aggregated Aβ species, the thiophene backbone needs to contain at least five units.^30,32,48^ Furthermore, when a combination of tetramer q-FTAA and heptamer h-FTAA was used, an age-dependent structural alteration of Aβ plaques in transgenic mice could be observed as a variation in spectral signatures.^42^ More recently, in a similar manner, double staining with q-FTAA and h-FTAA on tissue sections from a cohort of AD patients including individuals affected by sporadic AD (sAD) as well as familial AD (fAD) showed that Aβ plaques cluster as clouds of conformational variants.^43^ Lately, structural modifications of the LCOs have been made to improve spectral separation of protein aggregates or to achieve protein-selective binding. By replacing the central thiophene unit in pentameric or heptameric LCOs with a benzothiadiazole (BTD) moiety, several of the resulting so-called D-A-D (donor–acceptor–donor) ligands showed an increase in the ability to spectrally distinguish distinct Aβ deposits.^39^ Combining the thiophene backbone with other chemical scaffolds has rendered ligands selective for Aβ^37^ or tau^46^ deposits in AD.

Herein, we have studied the features of Aβ deposits in brain tissue sections from individuals with sAD or fAD associated with the *PSEN1 A431E* mutation by applying a novel combination staining protocol based on the new generation of thiophene-based ligands, an Aβ-selective ligand, HS-276,^37^ and a D-A-D ligand, LL-1.^39^ The choice of ligands was mainly based on two previous observations. First, spectral analysis has shown that the emission maximum of HS-276 upon interacting with Aβ deposits in sAD samples is 460 nm, whereas the corresponding peak for LL-1 is 660 nm.^37,39^ Second, results have indicated that the binding mode of HS-276 to Aβ deposits in sAD might be different from that of LL-1.^37^ To show the proof of concept for this new staining protocol, fAD cases associated with the *PSEN1 A431E* mutation^49−51^ were selected since these cases showed the largest difference from sAD when analyzing the properties of aggregated Aβ species using a previously reported LCO-based staining protocol.^43^ Moreover, neuropathologically, *PSEN1 A431E* mutation carriers display a larger set of Aβ deposit types compared to sAD cases; in fact, while CWPs are the most frequently observed, mature cored Aβ plaques, diffuse plaques, and primitive plaques are also detected in various amounts.^28^ In the present study, we show that in brain tissue sections from sAD or fAD associated with the *PSEN1 A431E* mutation, the ligands were binding differentially to the Aβ plaques indicating a structural or biochemical difference of the deposits. To assess the biochemical variation between HS-276- or LL-1-positive deposits in fAD, the Aβ peptide content in the plaques was analyzed using mass spectroscopy imaging.

## Results

### Combination Staining Reveals Different Patterns of Ligand Labeling

**Figure 1:**
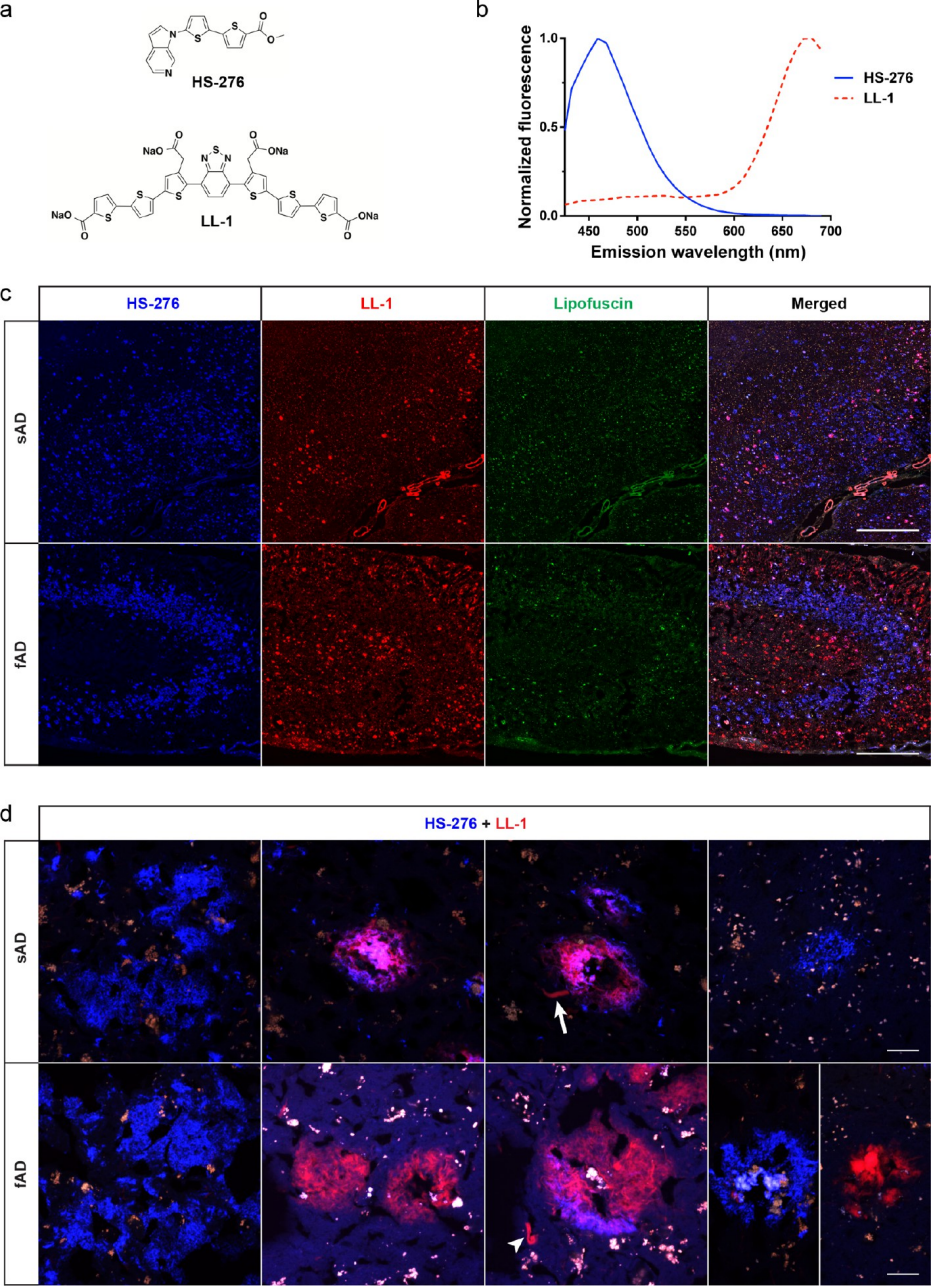
The combination protocol based on ligands HS-276 and LL-1 shows different staining patterns when applied on brain tissue sections from sAD and fAD (*PSEN1 A431E*) patients. (a) Chemical structures of ligands HS-276 (top) and LL-1 (bottom). (b) Fluorescence emission spectra of HS-276 (blue solid line) and LL-1 (red dashed line) when bound to Aβ-like structures in AD. (c) Fluorescence overview images of brain tissue sections from sAD (top panel) and fAD (bottom panel) patients stained with the combination of 200 nM HS-276 (blue) and 300 nM LL-1 (red). Autofluorescence from lipofuscin granules is shown in green. Scale bar, 1 mm. (d) Fluorescence images of different Aβ-like deposits in sAD (top panel) and fAD (bottom panel) brain tissue sections stained with the combination of HS-276 (blue) and LL-1 (red). LL-1 was also labeling structures resembling DNs (arrow) and NFTs (arrowhead). Autofluorescence from lipofuscin granules is shown in orange or in white. Scale bar, 20 μm.

The ligand binding mode to Aβ pathology in different types of AD was investigated by staining brain tissue sections (frontal cortex) from sporadic and familial cases with a combination of ligands HS-276 and LL-1 (Figure 1a). Samples representing the familial variant were associated with the *PSEN1 A431E* mutation. The combination staining of AD brain tissue sections showed that the blueshifted fluorescence emitted from HS-276 could easily be distinguished from the redshifted LL-1 emission (Figure 1b–d). In all AD brain samples, broad autofluorescence from intracellular lipofuscin granules^52,53^ could also be seen. To distinguish lipofuscin from ligand binding, an additional fluorescence channel was added in which the acquisition settings mainly allowed collection of autofluorescence. However, Aβ deposits exhibiting strong ligand fluorescence had some bleed-through in the lipofuscin channel, but since the morphology of the deposits was different compared to the lipofuscin granules, it was possible to distinguish ligand binding from autofluorescence (Figure 1c,d). To make it easier to compare ligand staining patterns, high-resolution images including a large field of view were obtained by subdividing the region of interest into smaller images that were then combined into an overview. These tile images showed that the staining patterns of the ligands were substantially different in sAD compared to fAD associated with the *PSEN1 A431E* mutation (Figure 1c and Figure S1). In sAD, HS-276 labeling was dominating, and several structures, particularly those resembling diffuse Aβ deposits, lacked or showed weak LL-1 positivity and were in most cases only stained with HS-276. In the white matter, assemblies of small dot-like structures solely labeled with HS-276 were also seen (Figure 1d). In contrast to HS-276, LL-1 single-stained Aβ-like aggregates were rarely found in the sAD brain tissue sections. LL-1-positive structures were observed in the parenchyma, but they always, besides a small number of diffuse morphologies in the gray matter, showed costaining with HS-276. These assemblies were densely packed and occasionally contained a core with high fluorescence signal from the ligands. LL-1, but not HS-276, was also staining densities resembling different types of tau pathologies such as dystrophic neurites (DNs). In DN-containing assemblies, both ligands were binding, indicating that LL-1, together with HS-276, labeled cored as well as neuritic Aβ plaques in sAD (Figure 1d). When applying the combination protocol on two additional sAD cases, the domination of HS-276 labeling was even more pronounced than the observation in the first case and LL-1 staining appeared to be limited to tau pathology (Figure S1a).

In the *PSEN1 A431E* cases, the main observation was the laminar staining pattern displayed by the ligands (Figure 1c and Figure S1). The result clearly showed that HS-276 and LL-1 were labeling structures in different cortical layers, with LL-1 positivity dominating in the outer and inner layers, whereas HS-276-stained deposits were found in the intermediate layers. HS-276-stained assemblies had an irregular shape similar to the structures denoted as diffuse plaques in sAD. Most of the LL-1-positive structures were round and compact and were lacking a dense core. In some of these assemblies, LL-1-labeled DNs could be seen, as well as varying levels of HS-276 infiltrations. LL-1 also stained assemblies similar to NFTs and neuropil threads. A small number of densities, resembling cored plaques, showed binding only to LL-1, while most of these structures were labeled with both LL-1 and HS-276 with the latter ligand dominating (Figure 1d). A laminar staining pattern was observed in two additional fAD *PSEN1 A431E* cases; however, in one of the samples, the layer of plaques that displayed HS-276 positivity also showed minor labeling with LL-1 (Figure S1b).

In the fAD, as well as in the sAD samples, ligand labeling of cerebral amyloid angiopathy (CAA) lesions in blood vessel walls could also be observed. In both AD types, the CAA pathology was mainly stained by LL-1, but in fAD, small sections of HS-276 positivity could occasionally be seen (Figure S2a). To examine the possibility of one ligand blocking the other from binding, the sAD and fAD *PSEN1 A431E* brain sections were also stained with each ligand by itself. The staining patterns were similar to those when performing the double-stain combination protocol, confirming that HS-276 and LL-1 may have distinct binding sites on the Aβ deposits (Figure S2b).

### Comparing Ligand Labeling in sAD and fAD (PSEN1 A431E) with Aβ Antibody Staining

**Figure 2:**
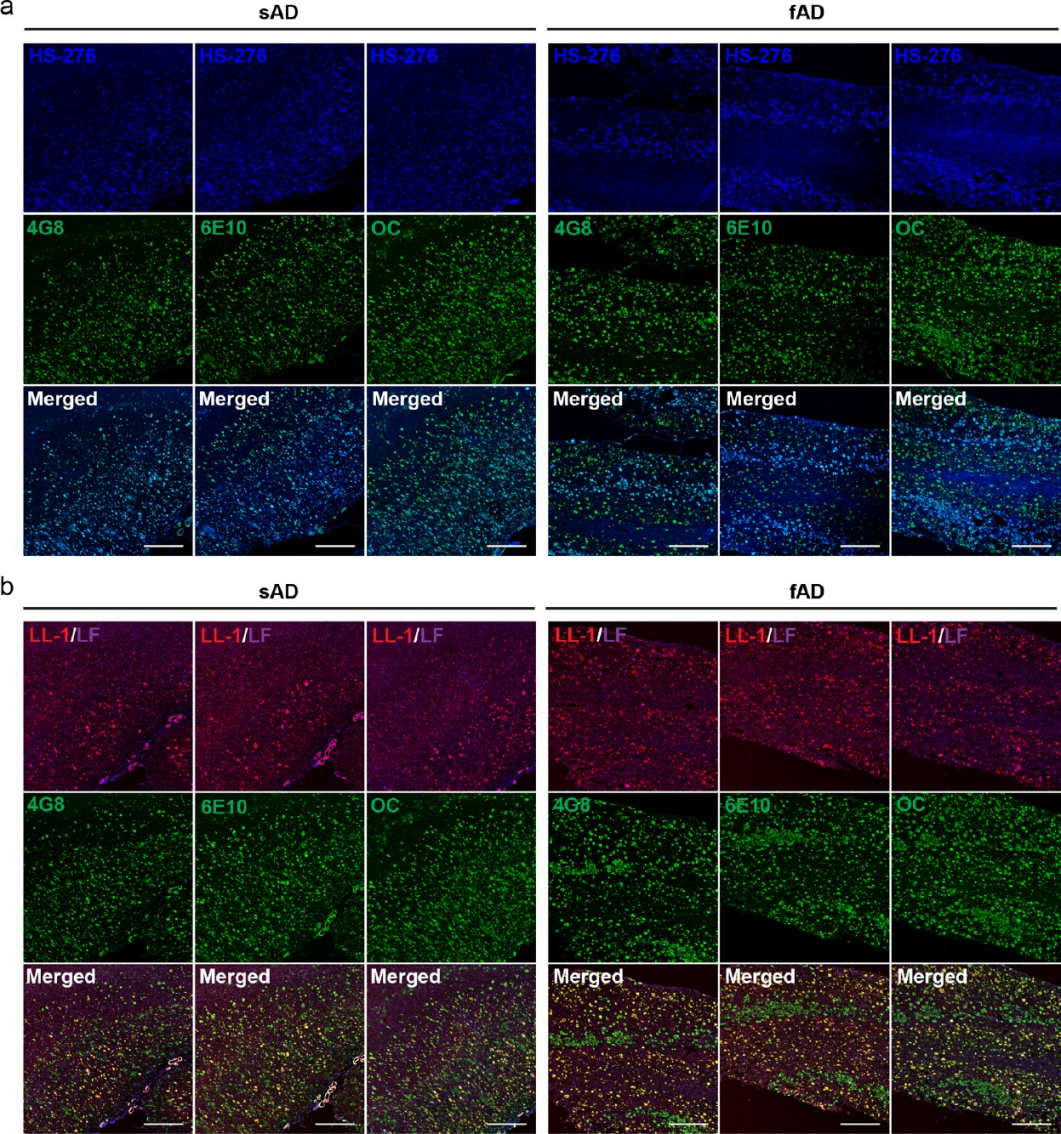
HS-276 and LL-1 label Aβ pathology in sAD and fAD (*PSEN1 A431E*) brain tissue sections. (a) Fluorescence overview images of tissue sections from sAD (left) and fAD (right) patients stained with 200 nM HS-276 (blue) and anti-Aβ antibody 4G8 (left panel, green), 6E10 (middle panel, green), or fibril specific antibody OC (right panel, green). Scale bar, 1 mm. (b) Fluorescence overview images of tissue sections from sAD (left) and fAD (right) patients stained with 300 nM LL-1 (red) and anti-Aβ antibody 4G8 (left panel, green), 6E10 (middle panel, green), or fibril specific antibody OC (right panel, green). Autofluorescence from lipofuscin (LF) is shown in purple. Scale bar, 1 mm.

**Figure 3:**
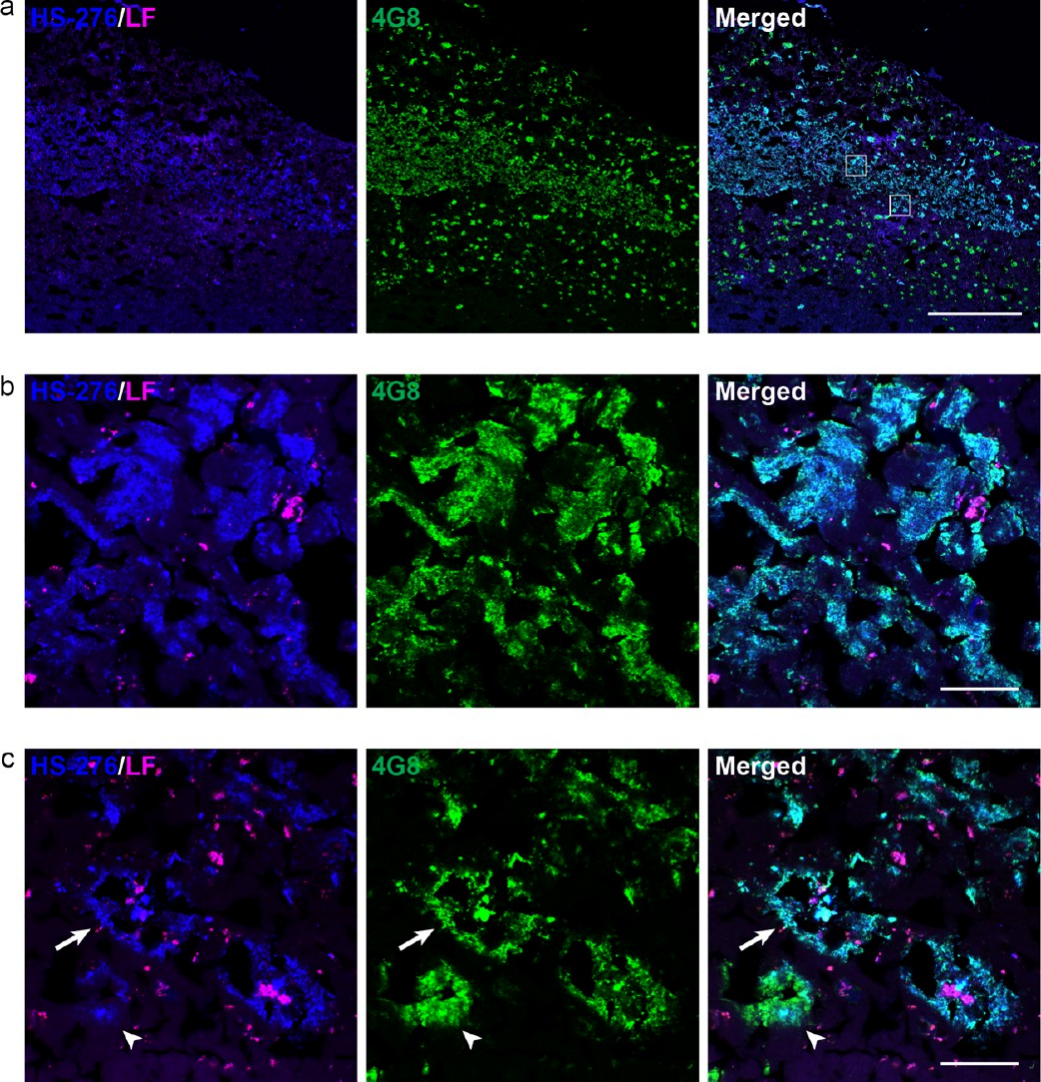
Parenchymal Aβ deposit types in fAD (*PSEN1 A431E*) labeled with HS-276. (a) Fluorescence overview image of brain tissue section from an fAD patient stained with 200 nM HS-276 (blue) and anti-Aβ antibody 4G8 (green). HS-276 mainly labels Aβ plaques in the intermediate cortical layers, whereas 4G8 stains deposits in all layers. Autofluorescence from lipofuscin (LF) granules is shown in magenta. The white boxes define the zoomed-in regions shown in (b) and (c). Scale bar, 1 mm. (b) Zoomed-in view of the top left region highlighted in (a) showing diffuse Aβ plaques labeled with HS-276 (blue) and 4G8 (green). Autofluorescence from lipofuscin (LF) granules is shown in magenta. Scale bar, 50 μm. (c) Zoomed-in view of the bottom right region highlighted in (a) showing cored Aβ plaques (arrow) labeled with HS-276 (blue) and 4G8 (green). In the outer parts of the HS-276-positive layer of Aβ plaques, deposits stained with 4G8, but only partially with HS-276, can be seen (arrowhead). Autofluorescence from lipofuscin (LF) granules is shown in magenta. Scale bar, 50 μm.

**Figure 4:**
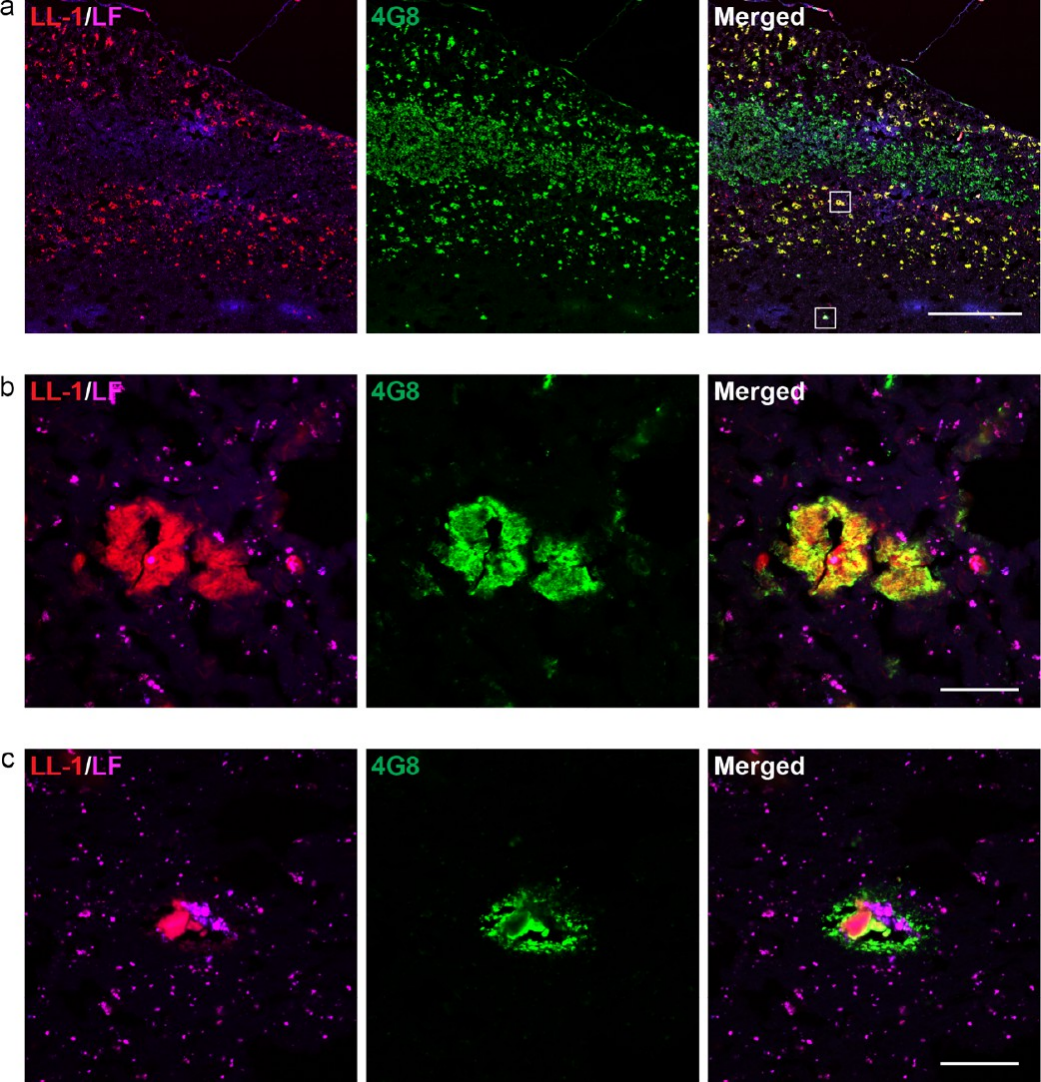
Parenchymal Aβ deposit types in fAD (*PSEN1 A431E*) labeled with LL-1. (a) Fluorescence overview image of brain tissue section from an fAD patient stained with 300 nM LL-1 (red) and anti-Aβ antibody 4G8 (green). LL-1 labels Aβ deposits in the inner and outer cortical layers, whereas the intermediate layers only show 4G8 positivity. The white boxes define the zoomed-in regions shown in (b) and (c). Scale bar, 1 mm. (b) Zoomed-in view of the top right region highlighted in (a) showing CWPs labeled with LL-1 (red) and 4G8 (green). Autofluorescence from lipofuscin (LF) granules is shown in magenta. Scale bar, 50 μm. (c) Zoomed-in view of the bottom left region highlighted in (a) showing cored Aβ plaque in the white matter labeled with LL-1. Autofluorescence from lipofuscin (LF) granules is shown in magenta. Scale bar, 50 μm.

When performing immunohistochemistry with antibodies directed against Aβ in combination with HS-276, the result showed that the ligand-positive structures in sAD were Aβ deposits, thereby corroborating earlier observations regarding HS-276 selectively binding to various types of Aβ pathologies in sAD brain tissue.^37^ Two Aβ antibodies, 6E10 and 4G8, and one pan-amyloid antibody, OC, were included in the study. In combination with antibodies 6E10 and 4G8, which have epitopes mapped to residues 5–7 and 17–21 of the Aβ peptide,^54^ respectively, the colocalization with HS-276 seemed to be complete. HS-276-positive deposits were also labeled by the OC antibody, which recognizes fibrils but not prefibrillar oligomers^55^ (Figure 2a). Due to bleed-through between the fluorescence channels, it was not possible to combine both ligands and antibodies on the same tissue section to investigate the double-stained structures found in the sAD tissue, but samples stained with only LL-1 together with the 6E10, 4G8, or OC antibody confirmed that this ligand also labels Aβ plaques (Figure 2b). However, the amount of Aβ deposits stained with LL-1 was significantly less than what was observed with HS-276. In the fAD cases, which were associated with the *PSEN1 A431E* mutation, the majority of structures were labeled with either HS-276 or LL-1. They displayed a distinct separation into different layers in the tissue, and when the ligands were applied together with the 6E10, 4G8, or OC antibody, the result showed that the ligand-positive assemblies in each layer were composed of Aβ (Figure 2a,b). Hence, in the *PSEN1 A431E* brain tissue, the areas containing most of the Aβ pathology displayed a clear variation in ligand binding, and the different plaque types accumulated in different layers resulting in a laminar staining pattern of the ligands. When examining the Aβ plaque types in each layer (Figure 3a), it was confirmed that HS-276 was binding to rather large and irregularly shaped Aβ deposits resembling diffuse plaques in the intermediate cortical layers (Figure 3b), as well as to a small number of cored plaques (Figure 3c). In addition, at the border of the HS-276-positive layer, Aβ deposits showing partial colocalization with the ligand and the 4G8 antibody were observed (Figure 3c) probably corresponding to the LL-1-labeled plaques with HS-276 infiltrations in Figure 1d. As mentioned above, LL-1 staining was only observed in the outer and inner cortical layers (Figure 4a). The majority of the LL-1-positive Aβ deposits were rather compact and lacked a dense core (Figure 4b). Since CWPs can be found in fAD and have previously been reported to be the most abundant type of Aβ deposit in examined *PSEN1 A431E* cases,^28^ the compact plaques labeled with LL-1 were probably CWPs. LL-1 was also binding to a small number of cored Aβ plaques (Figure 4c). The ligand-positive assemblies found in the blood vessel walls in sAD as well as in *PSEN1 A431E* sections, mainly labeled by LL-1, also showed staining with all included antibodies (Figure 2a,b).

### Comparing the Combination Staining Protocol in fAD (*PSEN1 A431E*) with Binding of Conventional Ligands

**Figure 5:**
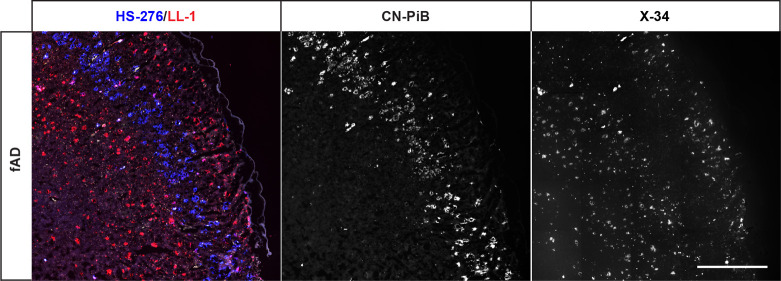
Fluorescence overview images of consecutive brain sections from an fAD (*PSEN1 A431E*) patient stained with the combination (left) of 200 nM HS-276 (blue) and 300 nM LL-1 (red) or with 200 or 300 nM of the conventional ligand CN-PiB (middle, white) or X-34 (right, white), respectively. CN-PiB labeled the same layer of Aβ plaques as HS-276, whereas the staining with X-34 corresponded to the staining pattern of LL-1. Scale bar, 1 mm.

Since fAD brain tissue sections from *PSEN1 A341E* mutation carriers showed a distinct laminar staining pattern resulting from HS-276 and LL-1 labeling separate layers of Aβ deposits (Figure 1c and Figure S1b), we next analyzed the binding properties of some conventional ligands to these layers. In a recent study, PiB was shown to underestimate the plaque burden in *PSEN1* cases containing CWPs due to its limited ability to detect this plaque type.^26^ To investigate if there was a correlation between ligand binding and PiB positivity, consecutive *PSEN1 A431E* brain tissue sections were stained either with the ligand combination, HS-276 and LL-1, or CN-PiB, a fluorescent structural analogue of PiB (Figure S3).^56^ The result clearly showed that CN-PiB was labeling the same layer of Aβ plaque as HS-276, whereas the regions demonstrating LL-1 positivity on the combined ligand section did not display any staining with the PiB analogue (Figure 5). Furthermore, when applying the fluorescent Congo red derivative X-34 (Figure S3)^57^ on the *PSEN1 A431E* brain tissue section consecutive to the one labeled with the ligand combination, it was shown to stain the same Aβ layers as LL-1 (Figure 5). Hence, in the *PSEN1 A431E* brain tissue, the Aβ binding properties of HS-276 were similar to those of the CN-PiB scaffold, whereas LL-1 interactions were comparable to the result displayed by X-34. Therefore, by applying the ligand combination instead of CN-PiB or X-34, a wider range of Aβ aggregates can be detected, and, in addition, since HS-276 and LL-1 display distinct colors when binding, the different types of Aβ plaques can be spectrally identified.

### Comparing the Combination Staining Protocol in fAD (*PSEN1 A431E*) with Binding of the Heptameric LCO h-FTAA

**Figure 6:**
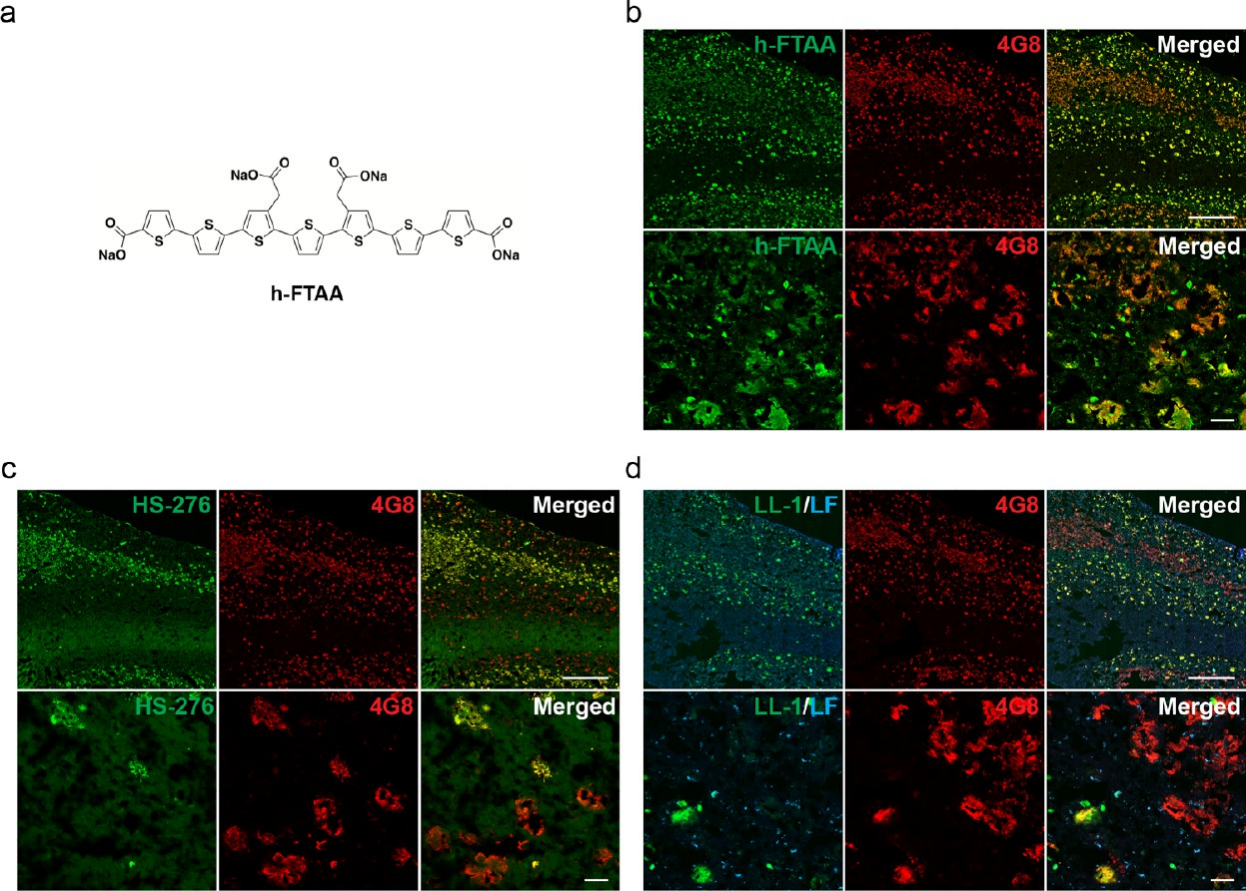
Comparing the combination staining protocol in fAD (*PSEN1 A431E*) with binding of LCOs and examining the effect on the staining result when increasing the concentration of HS-276 or LL-1. (a) Chemical structure of the LCO ligand h-FTAA. (b) Fluorescence images of fAD brain tissue section stained with h-FTAA (green) and anti-Aβ antibody 4G8 (red). An overview of the staining result is depicted in the top image, whereas the bottom image shows the binding pattern in more detail. H-FTAA showed complete colocalization with the antibody, confirming that the LCO ligand was binding to all Aβ deposits in the sample and not just specific types of plaques as HS-276 and LL-1. Scale bars, 1 mm (top panel) and 50 μm (bottom panel). (c,d) Fluorescence images of brain tissue section from fAD patient stained with 2 μM HS-276 (green) and anti-Aβ antibody 4G8 (red) (c) or 1 μM LL-1 (green) and anti-Aβ antibody 4G8 (red) (d). Overviews of the staining result are depicted in the top panels, whereas the bottom panels show the binding patterns in more detail. Even at the higher concentrations, both ligands were still mainly staining the same type of Aβ plaque as at the lower concentrations used in the combination protocol. Scale bars, 1 mm (top panel) and 50 μm (bottom panel).

In comparison with the chemical structure of HS-276 and LL-1, the conjugated backbone of the heptameric LCO h-FTAA only contains thiophene units (Figure 6a). h-FTAA has earlier shown superior binding to protein aggregates both in tissue sections and in vivo;^32,42,43,47^ therefore, we next investigated the staining pattern of this ligand in *PSEN1 A431E* brain tissues. When h-FTAA was applied on the *PSEN1 A431E* brain sections together with the anti-Aβ antibody 4G8, the analysis revealed a complete colocalization between the ligand and the antibody (Figure 6b). Hence, h-FTAA did not discriminate between the distinct layers of Aβ aggregates that were observed with the combination protocol but was labeling both HS-276- and LL-1-positive Aβ structures. The result indicates that the chemical structure of h-FTAA interacts differently with the Aβ deposits compared to the structures of HS-276 and LL-1. To investigate if the differences in ligand binding patterns were caused by variation in affinity, *PSEN1 A431E* brain tissue sections were stained with a 10 times higher concentration of HS-276 and a 3.3 times higher concentration of LL-1 than were used in the combination protocol. However, despite the 10-fold increase in the concentration, HS-276 only labeled the same layer of Aβ plaques that was observed when performing the combination staining (Figures 1c, 3b, and 6c), verifying that the binding site for the ligand is lacking on CWPs. With the higher concentration of LL-1, weak fluorescence could be seen from the diffuse deposits in the middle cortical layers not labeled with the ligand when using the combination protocol (Figures 1c, 3b, and 6d). The result suggests that the LL-1 can bind to this type of diffuse Aβ plaque (Figures 3b and 6d) but that its affinity for these deposits is significantly lower than for the aggregates observed with the combination protocol (Figure 4b).

### Tau Antibody Staining in fAD (*PSEN1 A431E*)

**Figure 7:**
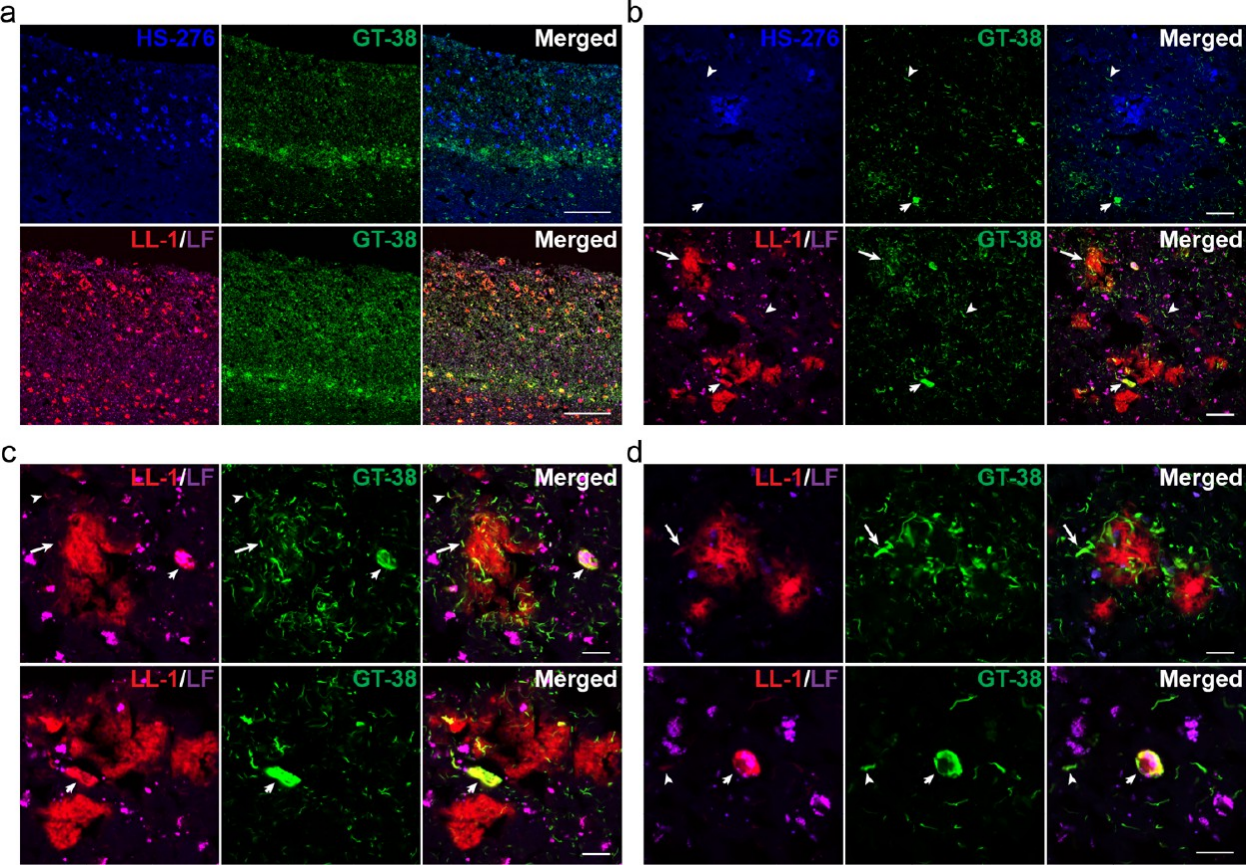
Distribution of tau pathology in fAD (*PSEN1 A431E*) brain tissue sample. (a) Fluorescence overview images of fAD brain sections stained with 200 nM HS-276 (top panel, blue) or 300 nM LL-1 (bottom panel, red) together with anti-tau antibody GT-38 (green). There is a marked increase in tau deposits accumulating just below the layer with HS-276-positive Aβ plaques. The pathology was extending into the upper part of the inner LL-1 labeled layer. Scale bar, 500 μm. (b) Fluorescence images showing the staining results in panel (a) in more detail. In many CWPs localized in the upper part of the LL-1 layer, antibody staining of DNs (arrow) could be seen. LL-1, but not HS-276, showed costaining with the tau antibody also in NFTs (small arrow) and neuropil threads (arrowhead). Scale bar, 50 μm. (c) Fluorescence images showing the staining results in panel (b) in a higher magnification. In several of the CWPs, DNs positive for LL-1 (red) and the tau antibody (green) can be seen (top panel, arrow). The LL-1 ligand is also labeling immunopositive neuropil threads (top panel, arrowhead) and NFTs (top/bottom panel, small arrow). Scale bar, 20 μm. (d) Fluorescence images of sAD brain tissue section stained with 300 nM LL-1 (red) and anti-tau antibody GT-38 (green). LL-1 is labeling immunopositive DNs (top panel, arrow), neuropil threads (bottom panel, arrowhead), and NFTs (bottom panel, small arrow). Scale bar, 20 μm.

To further characterize the different layers of Aβ plaque types displaying distinct ligand binding properties in *PSEN1 A431E* brain tissue, we examined the presence of tau pathology by performing staining with HS-276 or LL-1 in combination with the GT-38 tau antibody (Figure 7). GT-38 is a conformation-selective antibody that binds specifically to tau aggregates in AD brain tissue.^58^ The result showed that LL-1, but not HS-276, was labeling tau filaments in NFTs, DNs, and neuropil threads (Figure 7a–c). The pathological tau accumulations were present in all cortical layers; however, there was a marked increase of tau deposits just below the HS-276-positive layer of plaques. The pathology was extending into the upper part of the inner LL-1-labeled layer, and in many of the dense plaques in this region, antibody staining of DNs could be seen (Figure 7b,c). LL-1 and GT-38 double staining of sAD cases confirmed that the ligand was labeling tau pathology also in this type of AD (Figure 7d).

### Biochemical Characterization of HS-276- and LL-1-Positive Aβ Plaques in fAD (*PSEN1 A431E*)

**Figure 8:**
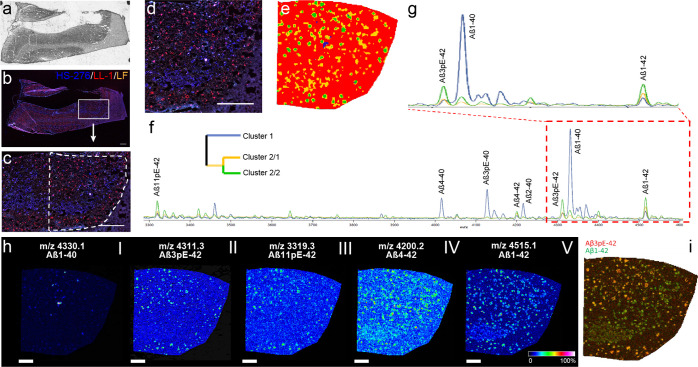
Correlative chemical imaging identifies HS-276 and LL-1 staining-associated Aβ deposition patterns. (a) Bright-field and (b–d) fluorescence microscopy ((c) and (d) are magnifications of (b)) images of fAD (*PSEN1 A431E*) frontal cortex brain tissue stained with HS-276 (blue) and LL-1 (red). Autofluorescent lipofuscin (LF) can be seen in orange. (e–i) MALDI MSI of Aβ peptides performed on brain section closely adjacent to the section labeled with HS-276 and LL-1. (e) Segmentation map using bisecting *k*-means cluster analysis (CA) identifies plaque-associated signatures following the plaque staining distribution patterns identified with HS-276 and LL-1. (f,g) Loading spectra and cluster tree of three spatial amyloid patterns retrieved by CA (1: blue, 2/1: yellow, and 2/2: green (e)). Inspecting the cluster-associated variable spectra shows the primary content within each cluster. Specifically, plaques within cluster 1 are dominated by Aβ*x*-40 peptides as further highlighted in the corresponding single ion map (h-I). Cluster 2 comprises a different pattern with two subtypes: 2/1 (e–g, yellow) and 2/2 (e–g, green). Plaques within cluster 2/2 show a significantly higher content of Aβ3pE-42 (h-II) and Aβ11pE-42 (h-III) and follow distribution of LL-1. In contrast, Aβ4-42 and Aβ1-42, while higher in cluster 2/2 (green), are also abundant in cluster 2/1 (yellow) as indicated by the single ion maps (h-IV and h-V) following HS-276 staining. (i) Overlay of Aβ3pE-42 and Aβ1-42 showing the complementary pattern outlined by CA (e), which is in line with LL-1/HS-276 staining (d). Scale bars, 1 mm (b–d) and 500 μm (h).

To determine the Aβ peptide content of the different plaque types identified with the combination staining protocol in *PSEN1 A431E* brain tissue sections, matrix-assisted laser desorption/ionization mass spectrometry imaging (MALDI MSI) was performed. This method enables comprehensive Aβ peptide analysis across individual Aβ plaques in situ.^59,60^ When comparing the acquired MALDI MSI data with the corresponding HS-276 and LL-1 staining patterns on closely adjacent brain sections (Figure 8a–g), the result revealed a correlation between Aβ peptide signatures and ligand binding (Figure 8h,i). By using bisecting *k*-means clustering-based image segmentation of the high-dimensional MALDI MSI data, the plaques could be divided into three distinct clusters based on the Aβ peptide content. In cluster 1, the deposits were dominated by Aβ*x*-40 peptides (Figure 8h-I). Cluster 2 showed different Aβ patterns and contained two subtypes, 2/1 and 2/2. Plaques in cluster 2/2 contained a significantly higher amount of amino-terminally truncated Aβ peptides ending at position 42 with pyroglutamate at position Glu-3 (Aβ3pE-42) or Glu-11 (Aβ11pE-42). These deposits correlated with the staining pattern of LL-1 (Figure 8h-II,h-III). Plaques in this cluster also showed a high content of Aβ4-42 and Aβ1-42. These peptides were also abundant in cluster 2/1, which followed the distribution of HS-276 labeling (Figure 8h-IV,h-V). When creating an overlay of Aβ3pE-42 and Aβ1-42, the complementary pattern of the cluster analysis was shown, which was in line with the staining of LL-1 and HS-276 (Figure 8i).

## Discussion

The development of ligand-based methods aimed at achieving specificity in the detection of Aβ plaques in the brain of AD patients would greatly facilitate the diagnosis of the disease, as well as aiding in assigning distinct aggregated proteinaceous species. PET tracers that bind to Aβ deposits have been introduced, but cases in which they fail to detect their target have been reported.^25,61^ A structural heterogeneity of Aβ filaments has been described both for in vitro and in vivo samples.^7−16^ This might explain the ligands’ inability to label certain types of Aβ assemblies. In earlier studies, the conformational-sensitive LCO ligands have been used to study Aβ deposits in AD transgenic mouse models as well as in AD brain samples.^34,42,43,62^ When applying the combination of q-FTAA and h-FTAA on APP/PS1 mouse brain tissue sections, the ligand staining patterns of the Aβ deposits were shown to be dependent on the age of the mouse.^42^ More recently, the ligand combination was applied on brain sections from patients diagnosed with distinct subtypes of AD, including sAD and cases of fAD associated with the *PSEN1 A431E* mutation.^43^ Since q-FTAA and h-FTAA display different emission profiles, the binding properties of each ligand could be assessed using hyperspectral imaging. When comparing the spectral signatures of all included Aβ deposits, the patient groups could be separated into distinct clouds. The subtypes, however, demonstrated a partial spectral overlap, and these LCOs, as shown on in vitro generated Aβ aggregates,^63^ most likely display a similar binding mode toward late formed Aβ assemblies. Thus, to assess different Aβ aggregates in a more refined manner, a combination of ligands with different binding modes toward Aβ deposits as well as distinct photophysical properties would be preferable. In the present study, we have introduced such a combination staining protocol based on ligands HS-276 and LL-1. The choice of ligands was the outcome of earlier observations indicating that HS-276 and LL-1 would display completely different emission profiles with emission peaks separated by 200 nm when binding to Aβ plaques, and, importantly, they have different binding modes toward these pathological entities.^37,38^ As fAD associated with the *PSEN1 A431E* mutation was the most different compared to other groups when using q-FTAA and h-FTAA staining,^43^ HS-276 and LL-1 staining was employed on brain tissue sections from sAD and *PSEN1 A431E* cases.

When the combination protocol was applied on sAD brain sections, strong labeling of HS-276 could be seen from cored, diffuse, and neuritic plaques in the gray matter as well as Aβ deposits in the white matter, whereas CAA lesions showed low or no HS-276 fluorescence intensity. A similar staining pattern for HS-276 on sAD brain sections has been described previously; however, in that protocol, only 100 nM HS-276 was used, which would explain the lack of ligand binding to CAA reported in that study.^37^ With LL-1, the number of labeled Aβ deposits was significantly less than with HS-276. Ligand binding could be seen from neuritic and cored plaques, which were also HS-276-positive, whereas diffuse plaques showed poor labeling. In the development of AD, diffuse plaques are considered being the first type of Aβ deposit that appears in the brain, whereas at later phases of Aβ deposition, other plaque types, such as cored and neuritic plaques, emerge.^64,65^ When examining diffuse plaques in ultrathin brain sections using electron microscopy, small amounts of Aβ filaments scattered between cell membranes could be seen.^66^ In addition, Fourier transform infrared imaging has shown that diffuse plaques mostly contained oligomeric and protofibrillar Aβ in low concentrations, whereas the cores of cored plaques displayed an abundance of filaments.^67^ This would explain the findings that diffuse deposits are negative for Congo red and only weakly stained with thioflavin S (ThS), whereas dense cored plaques are congophilic and show intense fluorescence when stained with ThS.^68,69^ We have previously reported that LCOs can be used to detect prefibrillar nonthioflavinophilic Aβ species.^48^ The ability to label early formed Aβ assemblies was dependent on the chemical structure of the ligand, and, for example, introducing sterically restricted moieties in the thiophene backbone has been shown to eliminate this property.^32,34^ In the LL-1 ligand, the central BTD unit is known to limit the flexibility of the backbone.^45^ Hence, this might explain why LL-1, and the rigid backbone found in the structure of Congo red, fails to bind to the reportedly less fibrillar content of diffuse plaques. From the CAA lesions, on the other hand, strong fluorescence from LL-1 could be observed. In contrast to parenchymal Aβ deposits, in which Aβ42 dominates, vascular deposits are mainly composed of Aβ40.^1−6^ Interestingly, in previous studies, it has been shown that plaque maturation is associated with an increase of the Aβ40 peptide.^59,70^ Hence, the combination protocol on sAD brain tissues indicates that LL-1 prefers binding to mature Aβ deposits containing Aβ40, whereas HS-276 binds to Aβ plaques mainly composed of the Aβ42 peptide.

When applying the double-stain combination protocol on brain sections from patients carrying the *PSEN1 A431E* mutation, the staining patterns of the ligands were different compared to what was observed in the sAD cases. In the *PSEN A431E* tissue, most of the immunopositive Aβ plaques were labeled with either HS-276 or LL-1. The ligands displayed a distinct laminar staining pattern with layers of round and dense LL-1-positive deposits surrounding a layer of irregularly shaped Aβ accumulations stained with HS-276, and different patterns of Aβ deposition have also been observed in other cases of fAD with different *PSEN1* or *APP* mutations.^71^ In the sAD cases, the dense type of Aβ plaque only labeled with LL-1 was not present, indicating that these LL-1-positive deposits in the *PSEN1 A431E* samples had a distinct structure and/or biochemical composition compared to the plaque types found in sAD. Further characterization showed that they were not binding to CN-PiB but to X-34. In a recent study, when these ligands were applied on brain tissue sections from different *PSEN1* mutation carriers, CWPs demonstrated identical staining properties to the LL-1-positive deposits.^26^ This, in combination with the round and dense configuration of the plaques, led us to conclude that LL-1 labeled CWPs in the *PSEN1 A431E* samples. CWPs have been described in fAD patients with various *PSEN1* mutations.^27,28,72−75^ Morphologically, in the electron microscope, the presence of Aβ fibrils can be found throughout the CWP, but the number can be small, and they are not forming compact cores.^74,75^ In addition, CWPs are often noncongophilic and show weak ThS fluorescence.^27^ Hence, although the fibrillar structure and the tinctorial properties of CWPs resemble the properties of diffuse plaques, they are still LL-1 positive, indicating that the formation of a binding site for this ligand does not require a dense arrangement of filaments. In the sAD cases, HS-276 showed robust labeling of all types of parenchymal Aβ deposits, but in the *PSEN1 A431E* samples, the ligand showed poor binding to the CWPs. Most of these deposits were HS-276-negative; however, in a small number of CWPs, minor infiltrations of HS-276 labeling could be seen. The HS-276-positive areas often occurred in CWPs that also displayed LL-1-labeled structures resembling DNs. In the *PSEN1 A431E* brain tissue, some of the CWPs demonstrated a high number of neurites, which, in addition to LL-1, also showed immunopositivity for tau. These plaques were often found in the cortical region that displayed the highest proportion of tau pathology. Regarding the regional accumulation of tau deposits, laminar distribution of tau deposits involving primarily cortical layers III and V has earlier been reported in AD.^76,77^ In contrast to the LL-1-stained CWPs, the HS-276-labeled Aβ structures were rather large and ill-limited with a speckled appearance. Histopathological characterization of the *PSEN1 A431E* mutation has previously revealed CWPs as the most abundant plaque type.^28^ However, the number of diffuse plaques was almost as high, which, in combination with the fact that they were most prevalent in the intermediate cortical layers,^28^ indicates that the HS-276-labeled Aβ deposits were of the diffuse type.

In *PSEN1 A431E* brain tissue, the LL-1-positive plaques showed binding to X-34, whereas HS-276 labeling corresponded to CN-PiB staining. Since it has previously been shown that LCOs compete for binding of X-34 but not PiB on AD brain-derived Aβ fibrils,^78^ we wanted to investigate their staining properties in the fAD cases. When applied on the *PSEN1 A431E* brain sections, the LCO h-FTAA showed complete colocalization with the Aβ antibody, confirming that this ligand was binding to LL-1- as well as HS-276-positive deposits. Hence, even though the staining results with HS-276 and LL-1 or CN-PiB and X-34 suggested that the Aβ plaques displayed a variation of ligand binding sites, h-FTAA was still able to bind all Aβ types underlining the pan-amyloid nature of h-FTAA. Recently, h-FTAA was also shown to label a larger variation of Aβ deposits in sAD brain tissue sections compared to LL-1.^38^ Structurally, the only difference between h-FTAA and LL-1 is the replacement of the central BTD unit in LL-1 with a thiophene moiety. The thiophene-only backbone is highly flexible and might therefore have an increased ability to adjust its conformation to fit into structurally diverse binding pockets on the Aβ fibrils. This is not achievable with the conformationally restricted LL-1 structure, which would explain the reduced ability of the ligand to detect certain types of Aβ deposits. Similar to LL-1, the X-34 ligand also has a more rigid structure compared to h-FTAA. This might prevent it from binding to the diffuse Aβ plaque types in the *PSEN1 A431E* samples. However, when used at higher concentrations, X-34 has been shown to label diffuse Aβ deposits in sAD,^57^ suggesting that the negative result in the *PSEN1 A431E* sample is due to lower affinity for this type of plaque. In fact, it has previously been demonstrated that the LCO backbone has higher affinity for protein aggregates than the Congo red scaffold.^33,41^ In the *PSEN1 A431E* tissue, HS-276 showed no labeling of the X-34-positive CWPs. Instead, the ligand was binding to the same type of Aβ plaque as CN-PiB, which has previously shown poor labeling of CWPs.^26^ Since increasing the ligand concentration by 10-fold did not change the staining results, the structure of the CWPs seems to lack a binding site for HS-276. It has already been reported that HS-276 does not share the same binding site on Aβ deposits as the D−A−D based LCO HS-169.^37^ Studies on synthetic Aβ filaments have shown that Congo red and PiB derivatives bind at distinct sites,^79^ suggesting that LL-1, h-FTAA, and X-34 might bind in the Congo red binding pocket, whereas HS-276 and CN-PiB bind at the PiB site, which seems to be lacking on the CWPs.

The binding mode of different ligands is most likely influenced by structural differences of the protein aggregates. From a structural perspective, the properties of the ligand binding pocket are dictated by the fold of the β-strands forming the general cross-β-sheet motif, and previous studies have shown that anionic LCOs bind to the same site as Congo red.^78,80^ Hence, the D-A-D-based LCO LL-1 is most likely interacting with repetitive lysine residues along the filament axis in a similar fashion to other LCOs. In contrast, according to the staining results, HS-276 might have a similar binding mode as PiB. However, further studies are necessary to pinpoint the exact binding mode of this ligand. Lately, cryo-EM structures of ligands bound to distinct protein aggregate filaments,^81,82^ as well as theoretical calculations using the folds obtained by cryo-EM studies,^83−85^ have shown that there are several different binding modes for amyloid ligands. Alternative binding modes of ligands can also be dependent on intermolecular interactions of protofilaments. For example, as mentioned previously, the LCO q-FTAA requires bundles of Aβ fibrils to bind, whereas h-FTAA interacts with solitary filaments.^63^ Moreover, biochemical modifications of the protein deposits can also alter the ligands’ ability to bind. For example, as shown by MALDI MSI, the LL-1-positive layers in *PSEN1 A431E* displayed Aβ deposits containing the pyroglutamate-modified Aβ peptides Aβ3pE-42 and Aβ11pE-42, which, in an earlier study, have been shown to be the main components of CWPs.^73^ In addition, the amounts of these peptides in the *PSEN1 A431E* Aβ deposits labeled with HS-276 were significantly lower. Hence, the observed differential binding mode of HS-276 and LL-1 to distinct Aβ deposits might be associated with a distinct biochemical composition of the aggregates. Whether these biochemical differences also render different structures of the Aβ deposits needs to be explored further with other techniques such as cryo-EM. In this regard, the dual-staining protocol with HS-276 and LL-1 might aid in isolating distinct Aβ deposits by laser capturing technologies.

Clearly, HS-276 and LL-1 can distinguish between different Aβ deposits in brain tissue sections, and it would of great interest to convert these ligands to PET tracers that can be employed for clinical diagnostics. For HS-276, such a transition is most likely possible since structurally related ligands, such as MK-6240,^86^ have been employed as a second-generation tau PET tracer. In contrast, earlier results^87^ have shown that oligothiophenes are not suitable as PET tracers due to poor brain uptake when used at low concentrations, as well as long duration time in the blood (2 weeks). Thus, although the oligothiophenes can be used for longitudinal optical in vivo imaging in transgenic mice,^29^ LL-1 and similar molecules cannot be converted into efficient PET tracers. On the other hand, due to cryo-EM structures obtained for different aggregated Aβ species,^15,16,88^ several alternative binding sites and novel molecular scaffolds more suitable for PET can be explored, and such studies are ongoing in our laboratory.

In conclusion, we have introduced a combination staining protocol based on the blueshifted ligand HS-276 and the redshifted ligand LL-1. When applied on brain tissue sections from patients diagnosed with sAD or fAD associated with the *PSEN1 A431E* mutation, labeling of Aβ pathology could be seen. In both types of AD, the Aβ plaques showed a variation in ligand staining patterns, indicating that distinct ligand binding sites are accessible on different types of Aβ plaques. Altogether, the results in this study prove that to be able to detect the entire spectrum of Aβ pathologies present in AD, a combination of ligands is required. Hence, a toolbox of PET tracers targeting distinct Aβ assemblies would most likely enhance the possibilities of an accurate diagnosis of AD, which is crucial to monitor disease progression, evaluate treatment strategies, and ultimately combat the disease.

## Materials and Methods

### Experimental Model and Subject Details

Frozen brain tissues from neuropathologically and genetically confirmed cases of sAD or fAD associated with the *PSEN1 A431E* mutation were obtained from the Dementia Laboratory at the Department of Pathology and Laboratory Medicine, Indiana University School of Medicine, Indianapolis, USA. The studies carried out at the Indiana University School of Medicine were reviewed and approved by the Indiana University Institutional Review Board, and informed consent was obtained from the patients or their next of kin. The experiments performed at Linköping University were reviewed and approved by a national ethical committee (approval number 2020-01197).

### Combination Staining with HS-276 and LL-1

HS-276 and LL-1 were synthesized as described previously.^37,39^ Frozen sections (10 μm) of the frontal cortex from three sAD and three fAD (*PSEN1 A431E* mutation) patients were fixed in 99.7% ethanol for 10 min and then rehydrated in 50% ethanol and dH_2_O. After incubation in phosphate-buffered saline (PBS, 10 mM phosphate, 140 mM NaCl, and 2.7 mM KCl, pH 7.4) for 10 min, the sections were stained with a combination of 200 nM HS-276^37^ and 300 nM LL-1^39^ for 30 min at RT. Sections stained only with 200 nM HS-276 or 300 nM LL-1 were also included. Excess ligands were removed by repeated washings with PBS. The sections were then mounted using a Dako mounting medium for fluorescence (Agilent). The result was analyzed using an inverted Zeiss 780 LSM confocal microscope (Zeiss) using the following excitation and emission settings: HS-276 exc 405 nm/em 415–527 nm; LL-1 exc 405 nm/em 599–703 nm; lipofuscin (autofluorescence) exc 405 nm/em 543–588 nm. The emission spectra of HS-276 and LL-1 when binding to Aβ deposits were collected on an inverted Zeiss 780 LSM confocal microscope (Zeiss) using an excitation wavelength of 405 nm.

### Ligand and Antibody Double Staining

Frozen frontal cortex brain sections (10 μm) from sAD and fAD (*PSEN1 A431E*) patients were fixed in prechilled acetone at −20 °C for 5 min and then allowed to dry for 30 min at RT. After a short step in PBS to remove the optimal cutting temperature (OCT) compound, the sections were incubated in PBS containing 5% normal goat serum (blocking buffer) for 30 min. The blocking buffer was then removed, and the primary antibody was added. For labeling of Aβ, antibodies 4G8 (Biolegend) and 6E10 (Biolegend) were used. To stain fibrils, the OC antibody (Merck) was employed, and for tau, the antibody GT-38 (Abcam) was used. All antibodies were diluted 1:1000 in the blocking buffer. After 2 h of incubation at RT, unbound antibodies were removed by washing in PBS for 3× 5 min. The tissue sections were then incubated with a goat anti-mouse or goat anti-rabbit secondary antibody conjugated with Alexa 488 (when in combination with LL-1) or Alexa 647 (when in combination with HS-276 or h-FTAA) for 1 h at RT. The secondary antibodies were diluted 1:400 in the blocking buffer. After washing in for PBS 3× 5 min, the sections were stained with 200 nM HS-276, 300 nM LL-1, or 200 nM h-FTAA, diluted in PBS, for 30 min at RT. The sections were then washed in PBS for 5 min and mounted with a Dako mounting medium for fluorescence (Agilent). The result was analyzed using an inverted Zeiss 780 LSM confocal microscope (Zeiss) exciting HS-276 and LL-1 at 405 nm, h-FTAA at 490 nm, Alexa 488 at 490 nm, and Alexa 647 at 633 or 640 nm.

### Staining with CN-PiB and X-34

Consecutive frozen frontal brain tissue sections (10 μm) from fAD (*PSEN1 A431E*) patient were fixed in 99.7% ethanol for 10 min, rehydrated, and then incubated in PBS as described above. The sections were stained with 200 nM CN-Pittsburgh compound-B (CN-PiB, a fluorescent analogue of the PiB PET tracer)^56^ or 300 nM X-34.^57^ All ligands were diluted in PBS. After 30 min of incubation, the sections were washed with PBS and mounted using a Dako mounting medium for fluorescence (Agilent). The staining pattern of each ligand was investigated using an inverted Zeiss 780 LSM confocal microscope (Zeiss) with the following excitation and emission settings: HS-276 exc 405 nm/em 415–527 nm; LL-1 exc 405 nm/em 599–703; lipofuscin (autofluorescence) exc 405 nm/em 543–588 nm; CN-PiB exc 405 nm/em 410–526 nm; X-34 exc 405 nm/em 410–526 nm.

### Matrix-Assisted Laser Desorption/Ionization Mass Spectrometry Imaging (MALDI MSI)

Brain tissue sections (10 μm) from a *PSEN1 A431E* mutation carrier were collected on a cryostat at an operating temperature of −15 °C. The sections were thaw mounted onto indium tin oxide (ITO)-coated, conductive glass slides (Bruker Daltonics) and stored at −20 °C until further use. Prior to matrix deposition, the sample was thawed in a desiccator under reduced pressure for 30 min. For amyloid peptide imaging, we employed a previously validated protocol for robust peptide and protein mass spectrometry imaging.^59^ A series of sequential washes of 100% EtOH (60 s), 70% EtOH (30 s), Carnoy’s fluid (6:3:1 EtOH/CHCl_3_/acetic acid) (90 s), 100% EtOH (15 s), H_2_O with 0.2% TFA (60 s), and 100% EtOH (15 s) was carried out for fixation, delipidation, and protein precipitation. The tissues were then subjected to formic acid vapor for 20 min.

A mixture of 2,5-dihydroxyacetophenone (2,5-DHAP) and 2,3,4,5,6-pentafluoroacetophenone (PFAP) matrix compounds was applied using an HTX TM-sprayer (HTX Technologies). A matrix solution of 5.7 μL/mL PFAP and 9.1 mg/mL DHAP in 70% ACN (aq) and 2% acetic acid/2% TFA was sprayed onto the tissue sections using the following instrumental parameters: a nitrogen flow of 10 PSI, a spray temperature of 75 °C, a nozzle height of 40 mm, eight passes with offsets and rotations, a spray velocity of 1000 mm/min, and an isocratic flow of 100 μL/min using 70% ACN as the pushing solvent.

MALDI MSI experiments were performed on a rapifleX Tissuetyper time-of-flight instrument (Bruker Daltonics). Measurements were performed at a 10 μm spatial resolution, at a laser pulse frequency of 10 kHz with 200 shots collected per pixel. Data were acquired in the linear positive mode in the mass range of 1500–6000 Da (mass resolution: *m*/Δ*m* = 1000 (fwhm) at *m*/*z* 4515). Preacquisition calibration of the system was performed using a combination of peptide calibration standard II and protein calibration standard I (Bruker Daltonics) in order to ensure calibration over the entire range of potential Aβ species. Image analysis of MSI data was performed in SCiLS (v 2021c, Bruker Daltonics). The data were interrogated by image segmentation using bisecting *k*-means clustering, implemented in SCiLS software.

To guide the MALDI MSI analysis of the *PSEN1 A431E* sample, a closely adjacent section was stained with the combination of 200 nM HS-276 and 300 nM LL-1 as described above. The fluorescence signal of each ligand was then spatially correlated with the MALDI MSI segmentation results and single ion maps.

## Supporting Information Available

Fluorescence images of additional sAD and fAD (*PSEN1 A431E*) cases stained with HS-276 and LL-1, fluorescence images showing CAA in sAD and fAD (*PSEN1 A431E*) labeled with HS-276 and LL-1, staining pattern of each ligand when used alone, and chemical structures of all included ligands (PDF)

## List of Abbreviations

Aβ: amyloid-β
Aβ3pE-42: amyloid-β peptide ending at position 42 with pyroglutamate at position Glu-3
Aβ11pE-42: amyloid-β peptide ending at position 42 with pyroglutamate at position Glu-11
AD: Alzheimer’s disease
BTD: benzothiadiazole
CAA: cerebral amyloid angiopathy
cryo-EM: cryogenic electron microscopy
CWPs: cotton wool plaques
D-A-D: donor–acceptor–donor
DHAP: dihydroxyacetophenone
DNs: dystrophic neurites
fAD: familial Alzheimer’s disease
LCOs: luminescent conjugated oligothiophenes
MALDI MSI: matrix-assisted laser desorption/ionization mass spectrometry imaging
NFTs: neurofibrillary tangles
PBS: phosphate-buffered saline
PET: positron emission tomography
PFAP: pentafluoroacetophenone
PiB: Pittsburgh compound-B
PSEN1: presenilin-1
sAD: sporadic Alzheimer’s disease
ThS: thioflavin S

## References

[ref1] Gkanatsiou E.; Portelius E.; Toomey C. E.; Blennow K.; Zetterberg H.; Lashley T.; et al. A distinct brain beta amyloid signature in cerebral amyloid angiopathy compared to Alzheimer’s disease. Neurosci. Lett. 2019, 701, 125–131. 10.1016/j.neulet.2019.02.033. 30807796

[ref2] Iwatsubo T.; Odaka A.; Suzuki N.; Mizusawa H.; Nukina N.; lharall Y. Visualization of Aβ42(43) and Aβ40 in senile plaques with end-specific Aβ monoclonals: evidence that an initially deposited species is Aβ42(43). Neuron 1993, 13, 45–53. 10.1016/0896-6273(94)90458-8. 8043280

[ref3] Joachim C. L.; Duffy L. K.; Morris J. H.; Selkoe D. J. Protein chemical and immunocytochemical studies of meningovascular β-amyloid protein in Alzheimer’s disease and normal aging. Brain Res. 1988, 474, 100–111. 10.1016/0006-8993(88)90673-7. 3214703

[ref4] Mann D. M. A.; Iwatsubo T.; Ihara Y.; Cairns N. J.; Lantos P. L.; Bogdanovic N.; et al. Predominant deposition of amyloid-β42(43) in plaques in cases of Alzheimer’s disease and hereditary cerebral hemorrhage associated with mutations in the amyloid precursor protein gene. Am. J. Pathol. 1996, 148, 1257–1266. 8644866 PMC1861527

[ref5] Miller D. L.; Papayannopoulos I. A.; Styles J.; Bobin S. A.; Lin Y. Y.; Biemann K.; Iqbal K. Peptide compositions of the cerebrovascular and senile plaque core amyloid deposits of Alzheimer’s disease. Arch. Biochem. Biophys. 1993, 301, 41–52. 10.1006/abbi.1993.1112. 8442665

[ref6] Prelli F.; Castano E.; Glenner G. G.; Frangione B. Differences between vascular and plaque core amyloid in Alzheimer’s disease. J. Neurochem. 1988, 51, 648–651. 10.1111/j.1471-4159.1988.tb01087.x. 3292706

[ref7] Gremer L.; Schölzel D.; Schenk C.; Reinartz E.; Labahn J.; Ravelli R. B. G.; et al. Fibril structure of amyloid-β(1–42) by cryo-electron microscopy. Science. 2017, 358, 116–119. 10.1126/science.aao2825. 28882996 PMC6080689

[ref8] Paravastu A. K.; Leapman R. D.; Yau W.-M.; Tycko R. Molecular structural basis for polymorphism in Alzheimer’s beta-amyloid fibrils. Proc. Natl. Acad. Sci. U. S. A. 2008, 105, 18349–18354. 10.1073/pnas.0806270105. 19015532 PMC2587602

[ref9] Petkova A. T.; Leapman R. D.; Guo Z.; Yau W.-M.; Mattson M. P.; Tycko R. Self-propagating, molecular-level polymorphism in Alzheimer’s β-Amyloid Fibrils. Science. 2005, 307, 262–265. 10.1126/science.1105850. 15653506

[ref10] Sachse C.; Fändrich M.; Grigorieff N. Paired beta-sheet structure of an Abeta(1–40) amyloid fibril revealed by electron microscopy. Proc. Natl. Acad. Sci. U. S. A. 2008, 105, 7462–7466. 10.1073/pnas.0712290105. 18483195 PMC2396686

[ref11] Schmidt M.; Rohou A.; Lasker K.; Yadav J. K.; Schiene-Fischer C.; Fändrich M.; et al. Peptide dimer structure in an Aβ(1–42) fibril visualized with cryo-EM. Proc. Natl. Acad. Sci. U.S.A. 2015, 112, 11858–11863. 10.1073/pnas.1503455112. 26351699 PMC4586870

[ref12] Wälti M. A.; Ravotti F.; Arai H.; Glabe C. G.; Wall J. S.; Böckmann A.; et al. Atomic-resolution structure of a disease-relevant Aβ(1–42) amyloid fibril. Proc. Natl. Acad. Sci. U.S.A. 2016, 113, E4976–84. 10.1073/pnas.1600749113. 27469165 PMC5003276

[ref13] Lu J.-X.; Qiang W.; Yau W.-M.; Schwieters C. D.; Meredith S. C.; Tycko R. Molecular structure of β-amyloid fibrils in Alzheimer’s disease brain tissue. Cell. 2013, 154, 1257–1268. 10.1016/j.cell.2013.08.035. 24034249 PMC3814033

[ref14] Qiang W.; Yau W.-M.; Lu J.-X.; Collinge J.; Tycko R. Structural variation in amyloid-β fibrils from Alzheimer’s disease clinical subtypes. Nature. 2017, 541, 217–221. 10.1038/nature20814. 28052060 PMC5233555

[ref15] Kollmer M.; Close W.; Funk L.; Rasmussen J.; Bsoul A.; Schierhorn A.; et al. Cryo-EM structure and polymorphism of Aβ amyloid fibrils purified from Alzheimer’s brain tissue. Nat. Commun. 2019, 10, 4760 10.1038/s41467-019-12683-8. 31664019 PMC6820800

[ref16] Yang Y.; Arseni D.; Zhang W.; Huang M.; Lövestam S.; Schweighauser S.; et al. Cryo-EM structures of amyloid-β 42 filaments from human brains. Science. 2022, 375, 167–172. 10.1126/science.abm7285. 35025654 PMC7612234

[ref17] Bateman R. J.; Xiong C.; Benzinger T. L. S.; Fagan A. M.; Goate A.; Fox N. C.; et al. Clinical and biomarker changes in dominantly inherited Alzheimer’s disease. N. Engl. J. Med. 2012, 367, 795–804. 10.1056/NEJMoa1202753. 22784036 PMC3474597

[ref18] Braak H.; Braak E. Diagnostic criteria for neuropathologic assessment of Alzheimer’s disease. Neurobiol. Aging. 1997, 18, 351–357. 10.1016/S0197-4580(97)00056-0. 9330992

[ref19] Jack C. R.; Lowe V. J.; Weigand S. D.; Wiste H. J.; Senjem M. L.; Knopman D. S.; et al. Serial PIB and MRI in normal, mild cognitive impairment and Alzheimer’s disease: implications for sequence of pathological events in Alzheimer’s disease. Brain. 2009, 132, 1355–1365. 10.1093/brain/awp062. 19339253 PMC2677798

[ref20] Jack C. R.; Knopman D. S.; Jagust W. J.; Shaw L. M.; Aisen P. S.; Weiner M. W.; et al. Hypothetical model of dynamic biomarkers of the Alzheimer’s pathological cascade. Lancet Neurol. 2010, 9, 119–128. 10.1016/S1474-4422(09)70299-6. 20083042 PMC2819840

[ref21] Price J. L.; Morris J. C. Tangles and plaques in nondemented aging and ″preclinical″ Alzheimer’s disease. Ann. Neurol. 1999, 45, 358–368. 10.1002/1531-8249(199903)45:3<358::AID-ANA12>3.0.CO;2-X. 10072051

[ref22] Klunk W. E.; Engler H.; Nordberg A.; Wang Y.; Blomqvist G.; Holt D. P.; et al. Imaging brain amyloid in Alzheimer’s disease with Pittsburgh Compound-B. Ann. Neurol. 2004, 55, 306–319. 10.1002/ana.20009. 14991808

[ref23] Ikonomovic M. D.; Klunk W. E.; Abrahamson E. E.; Mathis C. A.; Price J. C.; Tsopelas N. D.; et al. Post-mortem correlates of in vivo PiB-PET amyloid imaging in a typical case of Alzheimer’s disease. Brain. 2008, 131, 1630–1645. 10.1093/brain/awn016. 18339640 PMC2408940

[ref24] Svedberg M. M.; Hall H.; Hellström-Lindahl E.; Estrada S.; Guan Z.; Nordberg A.; et al. [11C] PIB-amyloid binding and levels of Aβ40 and Aβ42 in postmortem brain tissue from Alzheimer patients. Neurochem. Int. 2009, 54, 347–357. 10.1016/j.neuint.2008.12.016. 19162107

[ref25] Rosen R. F.; Ciliax B. J.; Wingo T. S.; Gearing M.; Dooyema J.; Lah J. J.; et al. Deficient high-affinity binding of Pittsburgh compound B in a case of Alzheimer’s disease. Acta Neuropathol. 2010, 119, 221–233. 10.1007/s00401-009-0583-3. 19690877 PMC3045810

[ref26] Abrahamson E. E.; Kofler J. K.; Becker C. R.; Price J. C.; Newell K. L.; Ghetti B.; et al. 11C-PiB PET can underestimate brain amyloid-β burden when cotton wool plaques are numerous. Brain. 2022, 145, 2161–2176. 10.1093/brain/awab434. 34918018 PMC9630719

[ref27] Crook R.; Verkkoniemi A.; Perez-Tur J.; Mehta N.; Baker M.; Houlden H.; et al. A variant of Alzheimer’s disease with spastic paraparesis and unusual plaques due to deletion of exon 9 of presenilin 1. Nat. Med. 1998, 4, 452–455. 10.1038/nm0498-452. 9546792

[ref28] Maarouf C. L.; Daugs I. D.; Spina S.; Vidal R.; Kokjohn T. A.; Patton R. L.; et al. Histopathological and molecular heterogeneity among individuals with dementia associated with Presenilin mutations. Mol. Neurodegener. 2008, 3, 20 10.1186/1750-1326-3-20. 19021905 PMC2600784

[ref29] Calvo-Rodriguez M.; Hou S. S.; Snyder A. C.; Dujardin S.; Shirani H.; Nilsson K. P. R.; et al. In vivo detection of tau fibrils and amyloid β aggregates with luminescent conjugated oligothiophenes and multiphoton microscopy. Acta Neuropathol. Commun. 2019, 7, 171 10.1186/s40478-019-0832-1. 31703739 PMC6839235

[ref30] Hammarström P.; Simon R.; Nyström S.; Konradsson P.; Åslund A.; Nilsson K. P. R. A fluorescent pentameric thiophene derivative detects in vitro-formed prefibrillar protein aggregates. Biochemistry. 2010, 49, 6838–6845. 10.1021/bi100922r. 20604540

[ref31] Johansson L. B. G.; Simon R.; Bergström G.; Eriksson M.; Prokop S.; Mandenius C.-F.; et al. An azide functionalized oligothiophene ligand-a versatile tool for multimodal detection of disease associated protein aggregates. Biosens. Bioelectron. 2015, 63, 204–211. 10.1016/j.bios.2014.07.042. 25089818

[ref32] Klingstedt T.; Åslund A.; Simon R. A.; Johansson L. B. G.; Mason J. J.; Nyström S.; et al. Synthesis of a library of oligothiophenes and their utilization as fluorescent ligands for spectral assignment of protein aggregates. Org. Biomol. Chem. 2011, 9, 8356–8370. 10.1039/c1ob05637a. 22051883 PMC3326384

[ref33] Klingstedt T.; Blechschmidt C.; Nogalska A.; Prokop S.; Häggqvist B.; Danielsson O.; et al. Luminescent conjugated oligothiophenes for sensitive fluorescent assignment of protein inclusion bodies. Chembiochem. 2013, 14, 607–616. 10.1002/cbic.201200731. 23450708 PMC3743175

[ref34] Klingstedt T.; Shirani H.; Mahler J.; Wegenast-Braun B. M.; Nyström S.; Goedert M.; et al. Distinct spacing between anionic groups: an essential chemical determinant for achieving thiophene-based ligands to distinguish β-amyloid or tau polymorphic aggregates. Chemistry. 2015, 21, 9072–9082. 10.1002/chem.201500556. 26013403 PMC4517144

[ref35] Klingstedt T.; Shirani H.; Åslund K. O. A.; Cairns N. J.; Sigurdson C. J.; Goedert M.; et al. The structural basis for optimal performance of oligothiophene-based fluorescent amyloid ligands: conformational flexibility is essential for spectral assignment of a diversity of protein aggregates. Chemistry. 2013, 19, 10179–10192. 10.1002/chem.201301463. 23780508 PMC3884759

[ref36] Klingstedt T.; Ghetti B.; Holton J. L.; Ling H.; Nilsson K. P. R.; Goedert M. Luminescent conjugated oligothiophenes distinguish between α-synuclein assemblies of Parkinson’s disease and multiple system atrophy. Acta Neuropathol. Commun. 2019, 7, 193 10.1186/s40478-019-0840-1. 31796099 PMC6892142

[ref37] Klingstedt T.; Shirani H.; Ghetti B.; Vidal R.; Nilsson P. R. Thiophene-based optical ligands that selectively detect Aβ pathology in Alzheimer’s disease. Chembiochem 2021, 22, 2568–2582. 10.1002/cbic.202100199. 34101954 PMC8409278

[ref38] Lantz L.; Shirani H.; Ghetti B.; Vidal R.; Klingstedt T.; Nilsson K. P. R. Thiophene-based ligands for histological multiplex spectral detection of distinct protein aggregates in Alzheimer′s disease. Chem. - Eur. J. 2023, 29, e202203568 10.1002/chem.202203568. 36645413 PMC10101888

[ref39] Lantz L.; Shirani H.; Klingstedt T.; Nilsson K. P. R. Synthesis and characterization of thiophene-based donor-acceptor-donor heptameric ligands for spectral assignment of polymorphic amyloid-β deposits. Chemistry. 2020, 26, 7425–7432. 10.1002/chem.201905612. 32022335 PMC7318160

[ref40] Magnusson K.; Simon R.; Sjölander D.; Sigurdson C. J.; Hammarström P.; Nilsson K. P. R. Multimodal fluorescence microscopy of prion strain specific PrP deposits stained by thiophene-based amyloid ligands. Prion. 2014, 8, 319–329. 10.4161/pri.29239. 25495506 PMC4601348

[ref41] Mahajan V.; Klingstedt T.; Simon R.; Nilsson K. P. R.; Thueringer A.; Kashofer K.; et al. Cross β-sheet conformation of keratin 8 is a specific feature of Mallory–Denk bodies compared with other hepatocyte inclusions. Gastroenterology. 2011, 141, 1080–1090. 10.1053/j.gastro.2011.05.039. 21699779

[ref42] Nyström S.; Psonka-Antonczyk K. M.; Ellingsen P. G.; Johansson L. B. G.; Reitan N.; Handrick S.; et al. Evidence for age-dependent in vivo conformational rearrangement within Aβ amyloid deposits. ACS Chem. Biol. 2013, 8, 1128–1133. 10.1021/cb4000376. 23521783

[ref43] Rasmussen J.; Mahler J.; Beschorner N.; Kaeser S. A.; Häsler L. M.; Baumann F.; et al. Amyloid polymorphisms constitute distinct clouds of conformational variants in different etiological subtypes of Alzheimer’s disease. Proc. Natl. Acad. Sci. U.S.A. 2017, 114, 13018–13023. 10.1073/pnas.1713215114. 29158413 PMC5724274

[ref44] Shahnawaz M.; Mukherjee A.; Pritzkow S.; Mendez N.; Rabadia P.; Liu X.; et al. Discriminating α-synuclein strains in Parkinson’s disease and multiple system atrophy. Nature 2020, 578, 273–277. 10.1038/s41586-020-1984-7. 32025029 PMC7066875

[ref45] Shirani H.; Linares M.; Sigurdson C. J.; Lindgren M.; Norman P.; Nilsson K. P. R. A palette of fluorescent thiophene-based ligands for the identification of protein aggregates. Chemistry. 2015, 21, 15133–15137. 10.1002/chem.201502999. 26388448 PMC4641461

[ref46] Shirani H.; Appelqvist H.; Bäck M.; Klingstedt T.; Cairns N. J.; Nilsson K. P. R. Synthesis of thiophene-based optical ligands that selectively detect tau pathology in Alzheimer’s disease. Chemistry. 2017, 23, 17127–17135. 10.1002/chem.201703846. 28926133 PMC5928317

[ref47] Wegenast-Braun B. M.; Skodras A.; Bayraktar G.; Mahler J.; Fritschi S. K.; Klingstedt T.; et al. Spectral discrimination of cerebral amyloid lesions after peripheral application of luminescent conjugated oligothiophenes. Am. J. Pathol. 2012, 181, 1953–1960. 10.1016/j.ajpath.2012.08.031. 23041059

[ref48] Åslund A.; Sigurdson C. J.; Klingstedt T.; Grathwohl S.; Bolmont T.; Dickstein D. L.; et al. Novel Pentameric thiophene derivatives for in vitro and in vivo optical imaging of a plethora of protein aggregates in cerebral amyloidosis. ACS Chem. Biol. 2009, 4, 673–684. 10.1021/cb900112v. 19624097 PMC2886514

[ref49] Murrell J.; Ghetti B.; Cochran E.; Macias-Islas M. A.; Medina L.; Varpetian A.; et al. The A431E mutation in PSEN1 causing Familial Alzheimer’s Disease originating in Jalisco state, Mexico: an additional fifteen families. Neurogenetics. 2006, 7, 277–279. 10.1007/s10048-006-0053-1. 16897084 PMC3378247

[ref50] Rogaeva E. A.; Fafel K. C.; Song Y. Q.; Medeiros H.; Sato C.; Liang Y.; et al. Screening for PS1 mutations in a referral-based series of AD cases: 21 novel mutations. Neurology. 2001, 57, 621–625. 10.1212/WNL.57.4.621. 11524469

[ref51] Yescas P.; Huertas-Vazquez A.; Villarreal-Molina M. T.; Rasmussen A.; Tusié-Luna M. T.; López M.; et al. Founder effect for the Ala431Glu mutation of the presenilin 1 gene causing early-onset Alzheimer’s disease in Mexican families. Neurogenetics. 2006, 7, 195–200. 10.1007/s10048-006-0043-3. 16628450

[ref52] Brunk U. T.; Terman A. Lipofuscin: mechanisms of age-related accumulation and influence on cell function. Free Radic. Biol. Med. 2002, 33, 611–619. 10.1016/S0891-5849(02)00959-0. 12208347

[ref53] Terman A.; Brunk U. T. Lipofuscin. Int. J. Biochem. Cell Biol. 2004, 36, 1400–1404. 10.1016/j.biocel.2003.08.009. 15147719

[ref54] Baghallab I.; Reyes-Ruiz J. M.; Abulnaja K.; Huwait E.; Glabe C.; Head E. Epitomic characterization of the specificity of the anti-amyloid Aβ monoclonal antibodies 6E10 and 4G8. J. Alzheimers Dis. 2018, 66, 1234–1244. 10.3233/JAD-180582. PMC6294585 30412489

[ref55] Kayed R.; Head E.; Sarsoza F.; Saing T.; Cotman C. W.; Necula M.; et al. Fibril specific, conformation dependent antibodies recognize a generic epitope common to amyloid fibrils and fibrillar oligomers that is absent in prefibrillar oligomers. Mol. Neurodegener. 2011, 2, 18 10.1186/1750-1326-2-18. PMC2100048 17897471

[ref56] Mathis C. A.; Wang Y.; Holt D. P.; Huang G.-F.; Debnath M. L.; Klunk W. E. Synthesis and evaluation of 11C-labeled 6-substituted 2-arylbenzothiazoles as amyloid imaging agents. J. Med. Chem. 2003, 46, 2740–2754. 10.1021/jm030026b. 12801237

[ref57] Styren S. D.; Hamilton R. L.; Styren G. C.; Klunk W. E. X-34, a fluorescent derivative of Congo red: a novel histochemical stain for Alzheimer’s disease pathology. J. Histochem. Cytochem. 2000, 48, 1223–1232. 10.1177/002215540004800906. 10950879

[ref58] Gibbons G. S.; Banks R. A.; Kim B.; Changolkar L.; Riddle D. M.; Leight S. N.; et al. Detection of Alzheimer disease (AD)-specific tau pathology in AD and nonAD tauopathies by immunohistochemistry with novel conformation-selective tau antibodies. J. Neuropathol. Exp. Neurol. 2018, 77, 216–228. 10.1093/jnen/nly010. 29415231 PMC6251598

[ref59] Michno W.; Nyström S.; Wehrli P.; Lashley T.; Brinkmalm G.; Guerard L.; et al. Pyroglutamation of amyloid-βx-42 (Aβx-42) followed by Aβ1–40 deposition underlies plaque polymorphism in progressing Alzheimer’s disease pathology. J. Biol. Chem. 2019, 294, 6719–6732. 10.1074/jbc.RA118.006604. 30814252 PMC6497931

[ref60] Michno W.; Wehrli P. M.; Blennow K.; Zetterberg H.; Hanrieder J. Molecular imaging mass spectrometry for probing protein dynamics in neurodegenerative disease pathology. J. Neurochem. 2019, 151, 488–506. 10.1111/jnc.14559. 30040875

[ref61] Schöll M.; Wall A.; Thordardottir S.; Ferreira D.; Bogdanovic N.; Långström B.; et al. Low PiB PET retention in presence of pathologic CSF biomarkers in Arctic APP mutation carriers. Neurology. 2012, 79, 229–236. 10.1212/WNL.0b013e31825fdf18. 22700814

[ref62] Liu H.; Kim C.; Haldiman T.; Sigurdson C. J.; Nyström S.; Nilsson K. P. R.; Cohen M. L.; et al. Distinct conformers of amyloid beta accumulate in the neocortex of patients with rapidly progressive Alzheimer’s disease. J. Biol. Chem. 2021, 297, 101267 10.1016/j.jbc.2021.101267. 34599965 PMC8531671

[ref63] Psonka-Antonczyk K. M.; Hammarström P.; Johansson L. B. G.; Lindgren M.; Stokke B. T.; Nilsson K. P. R.; et al. Nanoscale structure and spectroscopic probing of Aβ1–40 fibril bundle formation. Front. Chem. 2016, 4, 44 10.3389/fchem.2016.00044. 27921029 PMC5118468

[ref64] Thal D. R.; Rüb U.; Schultz C.; Sassin I.; Ghebremedhin E.; Del Tredici K.; et al. Sequence of Aβ-Protein Deposition in the Human Medial Temporal Lobe. J. Neuropathol. Exp. Neurol. 2000, 59, 733–748. 10.1093/jnen/59.8.733. 10952063

[ref65] Thal D. R.; Rüb U.; Orantes M.; Braak H. Phases of Aβ-deposition in the human brain and its relevance for the development of AD. Neurology. 2002, 58, 1791–1800. 10.1212/WNL.58.12.1791. 12084879

[ref66] Yamaguchi H.; Nakazato Y.; Hirai S.; Shoji M.; Harigaya Y. Electron micrograph of diffuse plaques. Initial stage of senile plaque formation in the Alzheimer brain. Am. J. Pathol. 1989, 135, 593–597. 2679112 PMC1880032

[ref67] Röhr D.; Boon B. D. C.; Schuler M.; Kremer K.; Hoozemans J. J. M.; Bouwman F. H.; et al. Label-free vibrational imaging of different Aβ plaque types in Alzheimer’s disease reveals sequential events in plaque development. Acta Neuropathol. Commun. 2020, 8, 222 10.1186/s40478-020-01091-5. 33308303 PMC7733282

[ref68] DeTure M. A.; Dickson D. W. The neuropathological diagnosis of Alzheimer’s disease. Mol. Neurodegener. 2019, 14, 32 10.1186/s13024-019-0333-5. 31375134 PMC6679484

[ref69] Ikeda S.; Allsop D.; Glenner G. G. A study of the morphology and distribution of amyloid beta protein immunoreactive plaque and related lesions in the brains of Alzheimer’s disease and adult Down’s syndrome. Prog. Clin. Biol. Res. 1989, 317, 313–323. 2532370

[ref70] Fukumoto H.; Asami-Odaka A.; Suzuki N.; Shimada H.; Ihara Y.; Iwatsubo T. Amyloid β protein deposition in normal aging has the same characteristics as that in Alzheimer’s disease. Predominance of Aβ42(43) and association of Aβ 40 with cored plaques. Am. J. Pathol. 1996, 148, 259–265. 10.1016/S0197-4580(96)80532-X. 8546214 PMC1861616

[ref71] Willumsen N.; Poole T.; Nicholas J. M.; Fox N. C.; Ryan N. S.; Lashley T. Variability in the type and layer distribution of cortical Aβ pathology in familial Alzheimer’s disease. Brain Pathol. 2022, 32, e13009 10.1111/bpa.13009. 34319632 PMC9048809

[ref72] Houlden H.; Baker M.; McGowan E.; Lewis P.; Hutton M.; Crook R.; et al. Variant Alzheimer’s disease with spastic paraparesis and cotton wool plaques is caused by PS-1 mutations that lead to exceptionally high amyloid-β concentrations. Ann. Neurol. 2000, 48, 806–808. 10.1002/1531-8249(200011)48:5<806::AID-ANA18>3.0.CO;2-F. 11079548

[ref73] Miravalle L.; Calero M.; Takao M.; Roher A. E.; Ghetti B.; Vidal R. Amino-terminally truncated Aβ peptide species are the main component of cotton wool plaques. Biochemistry. 2005, 44, 10810–10821. 10.1021/bi0508237. 16086583

[ref74] Takao M.; Ghetti B.; Hayakawa I.; Ikeda E.; Fukuuchi Y.; Miravalle L L.; et al. A novel mutation (G217D) in the Presenilin 1 gene (PSEN1) in a Japanese family: presenile dementia and parkinsonism are associated with cotton wool plaques in the cortex and striatum. Acta Neuropathol. 2002, 104, 155–170. 10.1007/s00401-002-0536-6. 12111359

[ref75] Verkkoniemi A.; Kalimo H.; Paetau A.; Somer M.; Iwatsubo T.; Hardy J.; et al. Variant Alzheimer disease with spastic paraparesis: neuropathological phenotype. J. Neuropathol. Exp. Neurol. 2001, 60, 483–492. 10.1093/jnen/60.5.483. 11379823

[ref76] Lemoine L.; Saint-Aubert L.; Nennesmo I.; Gillberg P.-G.; Nordberg A. Cortical laminar tau deposits and activated astrocytes in Alzheimer’s disease visualised by 3H-THK5117 and 3H-deprenyl autoradiography. Sci. Rep. 2017, 7, 45496 10.1038/srep45496. 28374768 PMC5379625

[ref77] Lewis D. A.; Campbell M. J.; Terry R. D.; Morrison J. H. Laminar and regional distributions of neurofibrillary tangles and neuritic plaques in Alzheimer’s disease: a quantitative study of visual and auditory cortices. J. Neurosci. 1987, 7, 1799–1808. 10.1523/JNEUROSCI.07-06-01799.1987. 2439665 PMC6568896

[ref78] Bäck M.; Appelqvist H.; LeVine H.; Nilsson K. P. R. Anionic oligothiophenes compete for binding of X-34 but not PIB to recombinant Aβ amyloid fibrils and Alzheimer’s disease brain-derived Aβ. Chemistry. 2016, 22, 18335–18338. 10.1002/chem.201604583. 27767229 PMC5215536

[ref79] Lockhart A.; Ye L.; Judd D. B.; Merritt A. T.; Lowe P. N.; Morgenstern J. L.; et al. Evidence for the presence of three distinct binding sites for the thioflavin T class of Alzheimer’s disease PET imaging agents on β-amyloid peptide fibrils. J. Biol. Chem. 2005, 280, 7677–7684. 10.1074/jbc.M412056200. 15615711

[ref80] König C.; Skånberg R.; Hotz I.; Ynnerman A.; Norman P.; Linares M. Binding sites for luminescent amyloid biomarkers from non-biased molecular dynamics simulations. Chem. Commun. (Camb). 2018, 54, 3030–3033. 10.1039/C8CC00105G. 29512664

[ref81] Shi Y.; Murzin A. G.; Falcon B.; Epstein A.; Machin J.; Tempest P.; et al. Cryo-EM structures of tau filaments from Alzheimer’s disease with PET ligand APN-1607. Acta Neuropathol. 2021, 141, 697–708. 10.1007/s00401-021-02294-3. 33723967 PMC8043864

[ref82] Merz G. E.; Chalkley M. J.; Tan S. K.; Tse E.; Lee J.; Prusiner S. B.; et al. Stacked binding of a PET ligand to Alzheimer’s tau paired helical filaments. Nat. Commun. 2023, 14, 3048 10.1038/s41467-023-38537-y. 37236970 PMC10220082

[ref83] Zou R.; Kuang G.; Ågren H.; Nordberg A.; Långström B.; Tu Y. Free energy profile for penetration of Pittsburgh compound-B into the amyloid β fibril. ACS Chem. Neurosci. 2019, 10, 1783–1790. 10.1021/acschemneuro.8b00662. 30698013

[ref84] Todarwal Y.; Gustafsson C.; Thi Minh N. N.; Ertzgaard I.; Klingstedt T.; Ghetti B.; et al. Tau protein binding modes in Alzheimer’s disease for cationic luminescent ligands. J. Phys. Chem. B 2021, 125, 11628–11636. 10.1021/acs.jpcb.1c06019. 34643404 PMC8558859

[ref85] Li J.; Kumar A.; Långström B.; Nordberg A.; Ågren H. Insight into the binding of first- and second-generation PET tracers to 4R and 3R/4R tau protofibrils. ACS Chem. Neurosci. 2023, 14, 3528–3539. 10.1021/acschemneuro.3c00437. 37639522 PMC10515481

[ref86] Walji A. M.; Hostetler E. D.; Selnick H.; Zeng Z.; Miller P.; Bennacef I.; et al. Discovery of 6-(fluoro-18F)-3-(1H-pyrrolo[2,3-c]pyridin-1-yl)isoquinolin-5-amine ([18F]-MK-6240): a positron emission tomography (PET) imaging agent for quantification of neurofibrillary tangles (NFTs). J. Med. Chem. 2016, 59, 4778–4789. 10.1021/acs.jmedchem.6b00166. 27088900

[ref87] Nordeman P.; Johansson L. B. G.; Bäck M.; Estrada S.; Hall H.; Sjölander D.; et al. 11C and 18F radiolabeling of tetra- and pentathiophenes as PET ligands for amyloid protein aggregates. ACS Med. Chem. Lett. 2016, 7, 368–373. 10.1021/acsmedchemlett.5b00309. 27096043 PMC4834648

[ref88] Yang Y.; Zhang W.; Murzin A. G.; Schweighauser M.; Huang M.; Lövestam S.; et al. Cryo-EM structures of amyloid-β filaments with the Arctic mutation (E22G) from human and mouse brains. Acta Neuropathol. 2023, 145, 325–333. 10.1007/s00401-022-02533-1. 36611124 PMC9925504

