# Supplementary material for: Thiophene-Based Ligands for Specific Assignment of Distinct Aβ Pathologies in Alzheimer's Disease
Source: ACS Chem Neurosci. 2024 Mar 25;15(7):1581–95. doi: 10.1021/acschemneuro.4c00021 (PMC10995944; doi:10.1021/acschemneuro.4c00021)
Supplement: Supplementary file 1 — cn4c00021_si_001.pdf [file cn4c00021_si_001.pdf]

Supporting Information

Thiophene-based ligands for specific assignment of distinct Aβ pathologies in Alzheimer´s disease

Therése Klingstedt[1], Linda Lantz[1], Hamid Shirani[1], Junyue Ge[2], Jörg Hanrieder[2,3], Ruben Vidal[4], Bernardino Ghetti[4], K. Peter R. Nilsson[1]*

[1]Department of Physics, Chemistry and Biology, Linköping University, 581 83 Linköping, Sweden

[2]Department of Psychiatry and Neurochemistry, Institute of Neuroscience and Physiology, The Sahlgrenska Academy, University of Gothenburg, Mölndal Hospital, 431 80 Mölndal, Sweden

[3]Department of Neurodegenerative Diseases, University College London Institute of Neurology, Queen Square, WC1N 3BG London. United Kingdom

[4]Department of Pathology and Laboratory Medicine, Indiana University School of Medicine, Indianapolis, Indiana 46202, USA

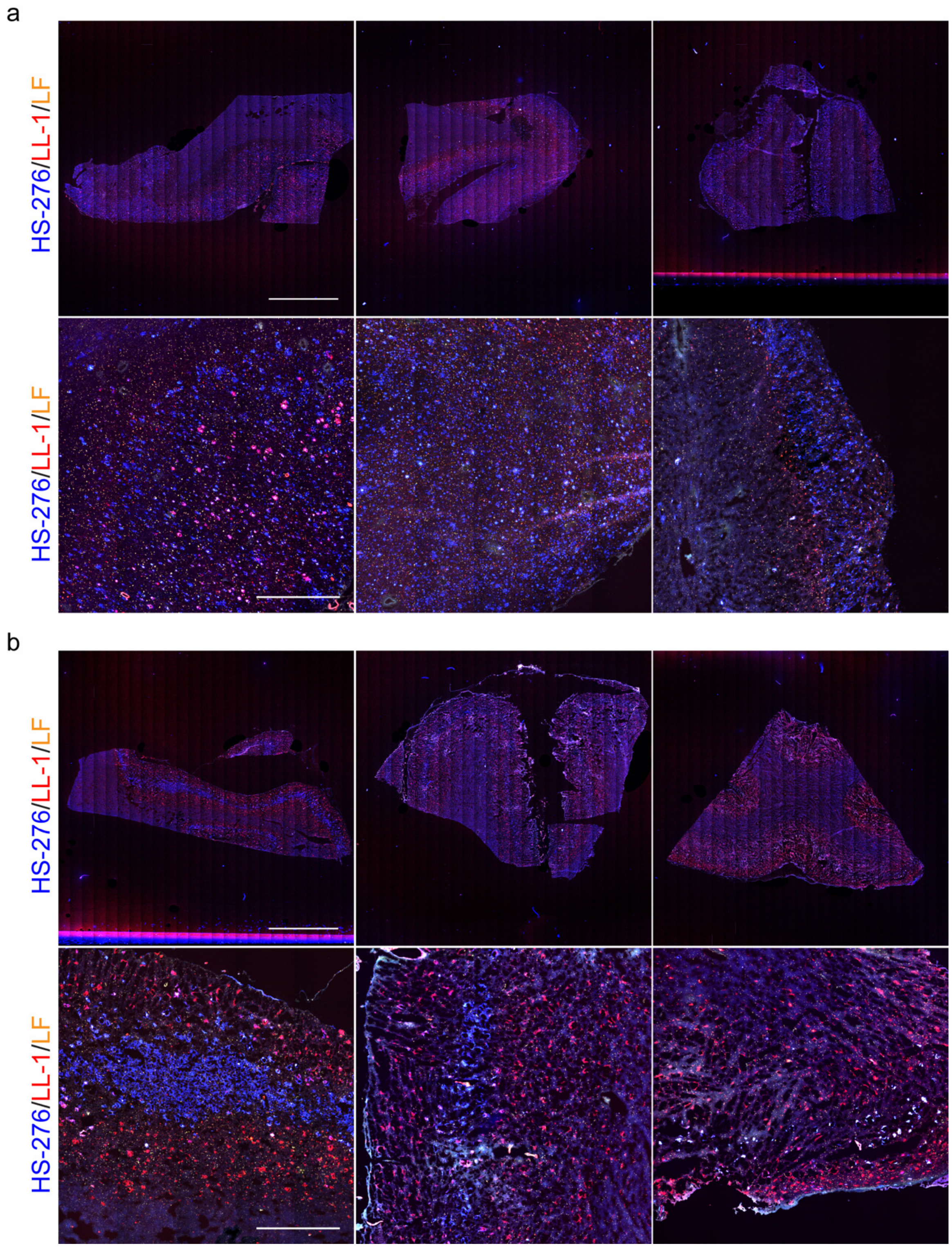

Figure S1. Using the ligand combination protocol on sAD and fAD (*PSEN1 A431E*) cases. **a** Fluorescence overview images of brain tissue sections from three different sAD cases stained with 200 nM HS-276 (blue) and 300 nM LL-1 (red). The images in the bottom panel were acquired at a higher magnification. The sAD case depicted on the left was used for the analysis shown in Fig 1, 2 and 7 and Suppl Fig 2. Autofluorescence from lipofuscin (LF) is shown in orange. Scale bars, 5 mm (top panel) and 1 mm (bottom panel). **b** Fluorescence overview images of brain tissue sections from three different fAD cases stained with 200 nM HS-276 (blue) and 300 nM LL-1 (red). The overview images in the bottom panel were acquired at a higher magnification. The fAD

case depicted on the left was used for the analysis shown in Fig 1, 2, 3, 4, 5, 6, 7 and 8 and Suppl Fig 2. Auto fluorescent LF granules are shown in orange. Scale bars, 5 mm (top panel) and 1 mm (bottom panel).

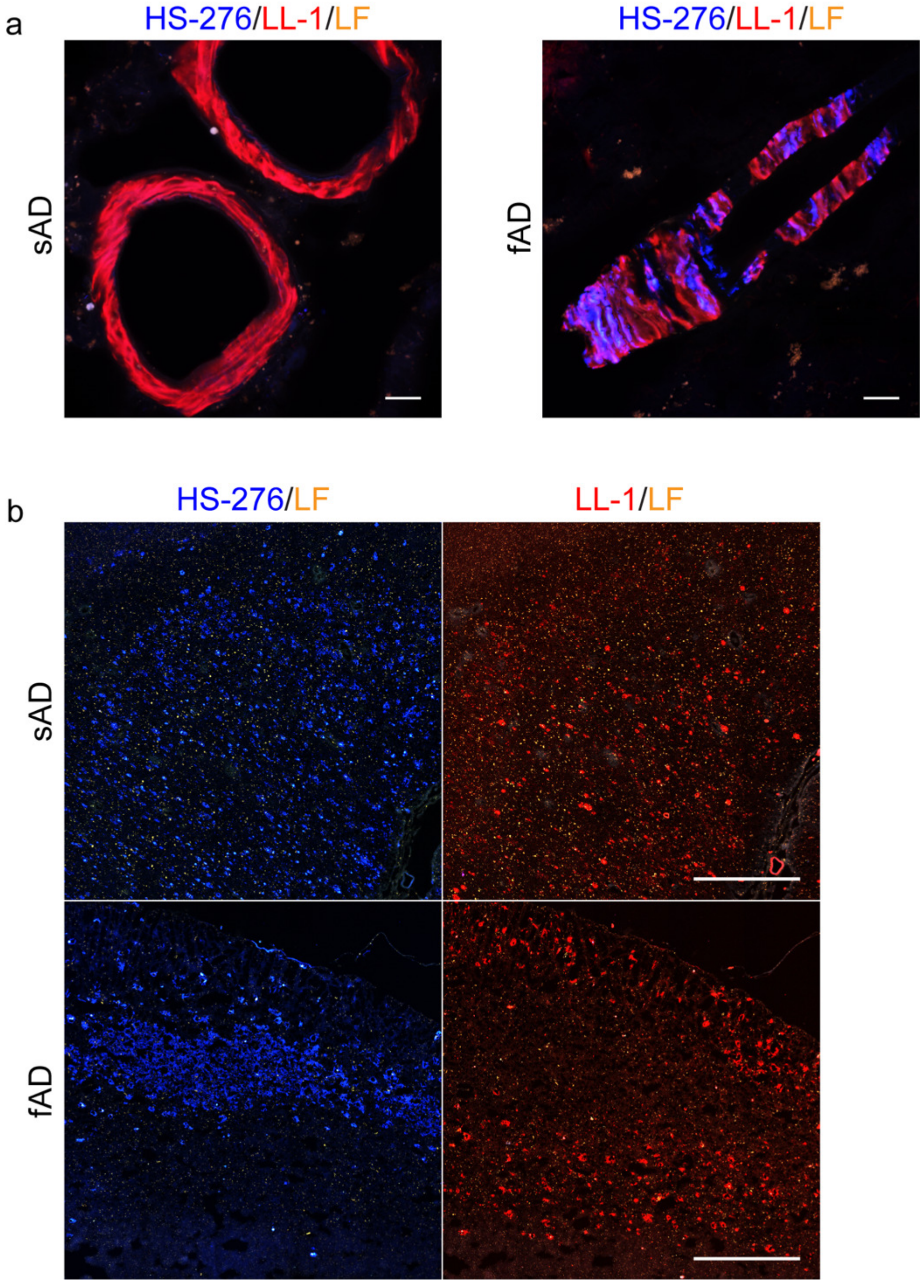

Figure S2. Characterization of the ligand combination protocol. **a** Fluorescence images of brain tissue section from sAD (left) or fAD (*PSEN1 A431E*, right) patient labelled with 200 nM HS-276 (blue) and 300 nM LL-1 (red) showing the labelling of CAA. Autofluorescence from lipofuscin (LF) is shown in orange. Scale bar, 20 µm. **b** Fluorescence overview images of brain tissue section from sAD (top panel) or fAD (*PSEN1 A431E*, bottom panel) patient stained with 200 nM HS-276 (blue) or 300 nM LL-1 (red). Autofluorescence from LF is shown in orange. Scale bar, 1 mm.

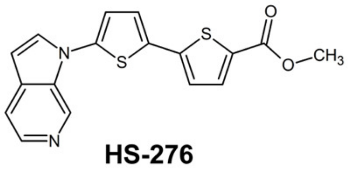

HS-276

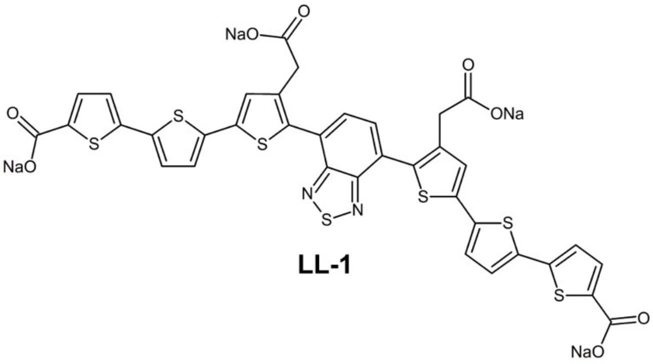

LL-1

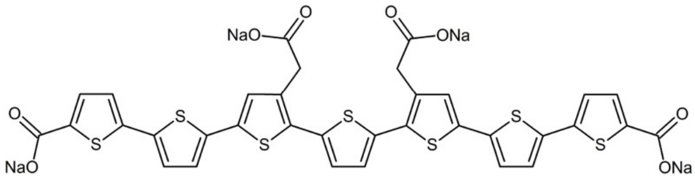

h-FTAA

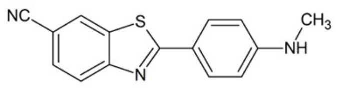

CN-PiB

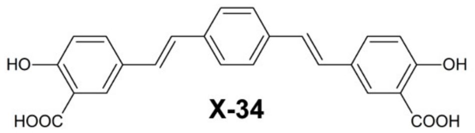

X-34

Figure S3. Chemical structures of ligands included in the study.
